# Nanobody screening and machine learning guided identification of cross-variant anti-SARS-CoV-2 neutralizing heavy-chain only antibodies

**DOI:** 10.1371/journal.ppat.1012903

**Published:** 2025-01-23

**Authors:** Peter R. McIlroy, Le Thanh Mai Pham, Thomas Sheffield, Maxwell A. Stefan, Christine E. Thatcher, James Jaryenneh, Jennifer L. Schwedler, Anupama Sinha, Christopher A. Sumner, Iris K. A. Jones, Stephen Won, Ryan C. Bruneau, Dina R. Weilhammer, Zhuoming Liu, Sean Whelan, Oscar A. Negrete, Kenneth L. Sale, Brooke Harmon

**Affiliations:** 1 Biotechnology and Bioengineering, Sandia National Laboratories, Livermore, California, United States of America; 2 Bioresource and Environmental Security, Sandia National Laboratories, Livermore, California, United States of America; 3 Biosecurity and Bioassurance, Sandia National Laboratories, Livermore, California, United States of America; 4 Systems Biology, Sandia National Laboratories, Livermore, California, United States of America; 5 Biosciences and Biotechnology Division, Lawrence Livermore National Laboratories, Livermore, California, United States of America; 6 Department of Molecular Microbiology, School of Medicine, Washington University, St. Louis, M issouri, United States of America; CharitΘ û University Medical School Berlin, Campus Benjamin Franklin, GERMANY

## Abstract

Severe Acute Respiratory Syndrome Coronavirus 2 (SARS-CoV-2) continues to persist, demonstrating the risks posed by emerging infectious diseases to national security, public health, and the economy. Development of new vaccines and antibodies for emerging viral threats requires substantial resources and time, and traditional development platforms for vaccines and antibodies are often too slow to combat continuously evolving immunological escape variants, reducing their efficacy over time. Previously, we designed a next-generation synthetic humanized nanobody (Nb) phage display library and demonstrated that this library could be used to rapidly identify highly specific and potent neutralizing heavy chain-only antibodies (HCAbs) with prophylactic and therapeutic efficacy *in vivo* against the original SARS-CoV-2. In this study, we used a combination of high throughput screening and machine learning (ML) models to identify HCAbs with potent efficacy against SARS-CoV-2 viral variants of interest (VOIs) and concern (VOCs). To start, we screened our highly diverse Nb phage display library against several pre-Omicron VOI and VOC receptor binding domains (RBDs) to identify panels of cross-reactive HCAbs. Using HCAb affinity for SARS-CoV-2 VOI and VOCs (pre-Omicron variants) and model features from other published data, we were able to develop a ML model that successfully identified HCAbs with efficacy against Omicron variants, independent of our experimental biopanning workflow. This biopanning informed ML approach reduced the experimental screening burden by 78% to 90% for the Omicron BA.5 and Omicron BA.1 variants, respectively. The combined approach can be applied to other emerging viruses with pandemic potential to rapidly identify effective therapeutic antibodies against emerging variants.

## Author summary

We leveraged a robust experimental pipeline for heavy chain-only (HCAb) library screening to identify 59 potent HCAbs that can cross-neutralize different SARS-CoV-2 variants. Several of these HCAbs with efficacy against different variants also bind to different SARS-CoV-2 epitopes, suggesting they could be used in antibody cocktails or be engineered as bispecific antibodies that are cross-variant and resistant to viral escape. Using existing published data and data generated from our library of HCAbs against diverse pre-Omicron SARS-CoV-2 variants, we developed an ML model to rapidly predict which HCAbs would be effective against Omicron BA.1 and Omicron BA.5. Using this ML model three HCAbs with efficacy against Omicron BA.1 and BA.5 were identified without requiring additional biopanning. This integrated computational and experimental pipeline can be leveraged for future outbreaks to rapidly identify cross-variant countermeasures that are effective against potential emerging variants.

## Introduction

The increased prevalence of worldwide outbreaks such as the 2002–2003 SARS-CoV outbreak, the 2009 H1N1 Influenza pandemic, and the ongoing SARS-CoV-2 pandemic warrants development of an integrated, high-throughput approach to identify medical countermeasures against emerging and re-emerging pathogens [1,2]. Current approaches, including vaccination, present challenges for timing, distribution, and generation of selective pressure against circulating strains. For example, during the SARS-CoV-2 pandemic, new variants iteratively emerged and established dominance due to their ability to escape vaccine-elicited antibodies [3]. Although many therapeutic antibodies against the viral spike protein have been identified, those that neutralize all variants of concern (VOCs) are rare [4–6]. Identifying antibodies with cross-variant reactivity, especially against different SARS-CoV-2 variants, is crucial to developing therapeutics that provide broad protection against future variants. Furthermore, a method for rapid identification of antibodies with efficacy against a wide range of potential viral variants provides a foundation for developing timely and effective therapeutics in response to emerging viruses with pandemic potential. Approaches combining experimental techniques and computational modeling will greatly improve our ability to quickly develop, test, and validate effective antibody therapeutics with maximum breadth and resistance to escape [7,8].

Several available antibody design tools use high-quality antibody/antigen co-complex structures or structures predicted from the antibody and antigen protein sequences [9]. However, structure-based methods are very time-consuming, limiting the number of viral variants and antibody designs that can be considered. For example, a previous study found that it took 1,250 CPU hours on a super computer to predict the three-dimensional structure of an antibody from its sequence using RosettaAntibody [10,11], and dock it to an antigen whose structure had already been computed using SnugDock [12]. While this may be feasible for low-throughput applications, it is not fast enough to address the numerous possible viral variants that may emerge.

The primary amino acid sequence remains the most accessible and complete type of antigen and antibody protein information. Consequently, many sequence-based feature extraction methods have been developed [13–17]. Existing machine learning (ML)-based prediction methods have several shortcomings: (i) They are often implemented for one specific antigen and therefore are not widely applicable; (ii) Most of the developed models are binary classifiers, only predicting whether an antibody–antigen pair is an ’interacting’ or a ’non-interacting’ pair; (iii) Models are usually evaluated through cross-validations and focus specifically on existing viral variants, which can lead to poor performance for emerging variants. Effective ML models using primary sequences as inputs, with the capability to predict antibody-antigen interactions without computationally expensive methods (e.g., those that first require structural predictions), would be a significant advancement in the field. In all cases, large amounts of relevant high-quality data are critical for training effective ML models to assist in the rational design of effective, escape-resistant antibody cocktails.

To address these gaps, we describe a strategy combining high-throughput screening of our high diversity humanized nanobody (Nb) library [18] against multiple variant spike receptor binding domains (RBDs) with computational modeling to identify cross-variant heavy chain only antibodies (HCAbs) that bind and neutralize divergent SARS-CoV-2 variants of interest (VOIs) and VOCs. Nbs are the variable antigen binding region of HCAbs found in camelids. Like conventional antibodies, Nbs and HCAbs bind their target antigen with high affinity but have a lower molecular weight, have greater solubility, higher thermostability, decreased production costs, and unique structural attributes that facilitate binding to antigen sites inaccessible to traditional IgG antibodies [18,19].

We initially screened our Nb library [18] against the RBDs from SARS-CoV-2 VOCs and VOIs that were circulating in the spring, summer, and fall of 2021. Nb sequences with affinity for these variant RBDs were produced as humanized HCAbs and were evaluated for binding affinity and neutralization potency against multiple VOIs and VOCs. To minimize screening efforts and identify cross-reactive HCAbs, we trained an ML model based on the binding affinities between HCAbs and SARS-CoV-2-RBD variants. We iteratively developed this model using pre-Omicron variant data and used it to predict the binding efficacy of our panel of HCAbs to the RBD of Omicron variants, with the model successfully predicting binding efficacy for Omicron BA.1, but not Omicron BA.5. To increase the accuracy of predictions for Omicron BA.5, data from HCAb binding to the Omicron BA.1 RBD was added to the training set, this resulted in an increase in the accuracy of binding predictions for Omicron BA.5. In this study, we present a comparative evaluation of broadly neutralizing HCAbs with efficacy against the original variant, VOIs, and VOCs, including the more recent Omicron variants BA.1 and BA.5. By panning against multiple variants of the RBD with distinct mutations and developing an ML model we successfully identified HCAbs with efficacy against existing and newly emerging variants.

## Results

### Potent HCAbs tightly bind SARS-CoV-2 RBD VOIs and VOCs

**Fig 1:**
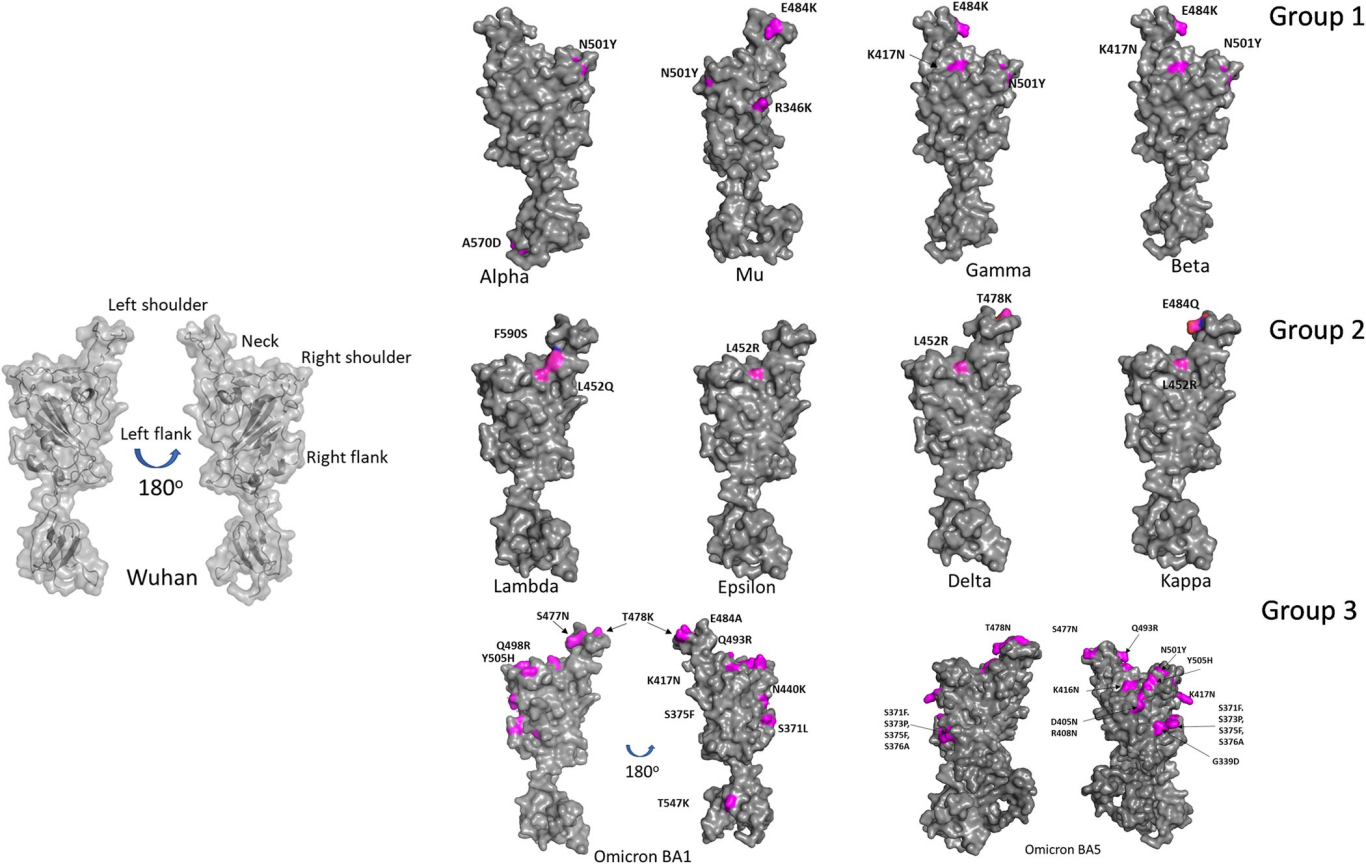
Location of amino acid substitutions in the spike protein of the original and SARS-Cov-2 VOIs and VOCs. Wild-type (WT)- PDB: 6VXX; Alpha- PDB: 7R14; Mu-PDB; Gamma- PDB: 7VX1; Beta-PDB: 7VX1; Delta- PDB: 7W92; Omicron–PDB: 7T9K; other variants, Lambda, Kappa, Epsilon and Mu structure were modeled by using Swiss-model server and WT (PDB: 6VXX) as a template. The structures were shown as surfaces, with RBD colored in gray and mutations highlighted in pink. Group 1 includes the RBDs from the Alpha, Beta, Gamma, and Mu SARS-CoV-2 variants, Group 2 includes the RBDs from Epsilon, Delta, Kappa, and Lambda variants and Group 3 includes the RBDs from Omicron BA.1 and Omicron BA.5.

To identify Nb sequences with affinity for SARS-CoV-2 VOIs and VOCs, our previously described synthetic humanized Nb phage display library was used to perform four rounds of selection against the RBD of the Alpha, Beta, Delta, Epsilon, Lambda, and Kappa SARS-CoV-2 variants [18]. To inform development of sequence-based ML models and highlight the potential for cross-variant efficacy, SARS-CoV-2 VOIs and VOCs were organized into three groups based on their common amino acid substitutions and mutation sites in the spike RBD **(Fig 1).** Within the Group 1 SARS-CoV-2 variants (Alpha, Beta, Gamma, & Mu), RBD mutations that potentially increase transmissibility and decrease the effectiveness of vaccines and therapeutics are prevalent. These include the E484K and N501Y mutations found in Beta (B.1.351) and Gamma (B.1.1.28) VOCs [20]. The E484K substitution has been shown to increase the number of serum antibodies needed to prevent infection of cells [21], while the N501Y substitution results in increased affinity of the viral spike protein for the angiotensin-converting enzyme 2 (ACE2) cellular receptor [22]. Within the Group 2 SARS-CoV-2 variants, which include the Delta, Kappa, Epsilon, and Lambda variants, the L452R/Q substitution on the neck of SARS-CoV-2 RBD led to higher infection rates of both vaccinated and unvaccinated people than previous VOIs and VOCs **(Fig 1)** [23–26]. The Delta variant, which also has the T478K mutation, potentially increased viral infectivity and raised concerns about vaccine efficacy. Here, we present a comparative evaluation of broadly neutralizing HCAbs with efficacy against the original variant, VOIs, and VOCs, including more recent Omicron variants BA.1 and BA.5 (Group 3).

**Fig 2:**
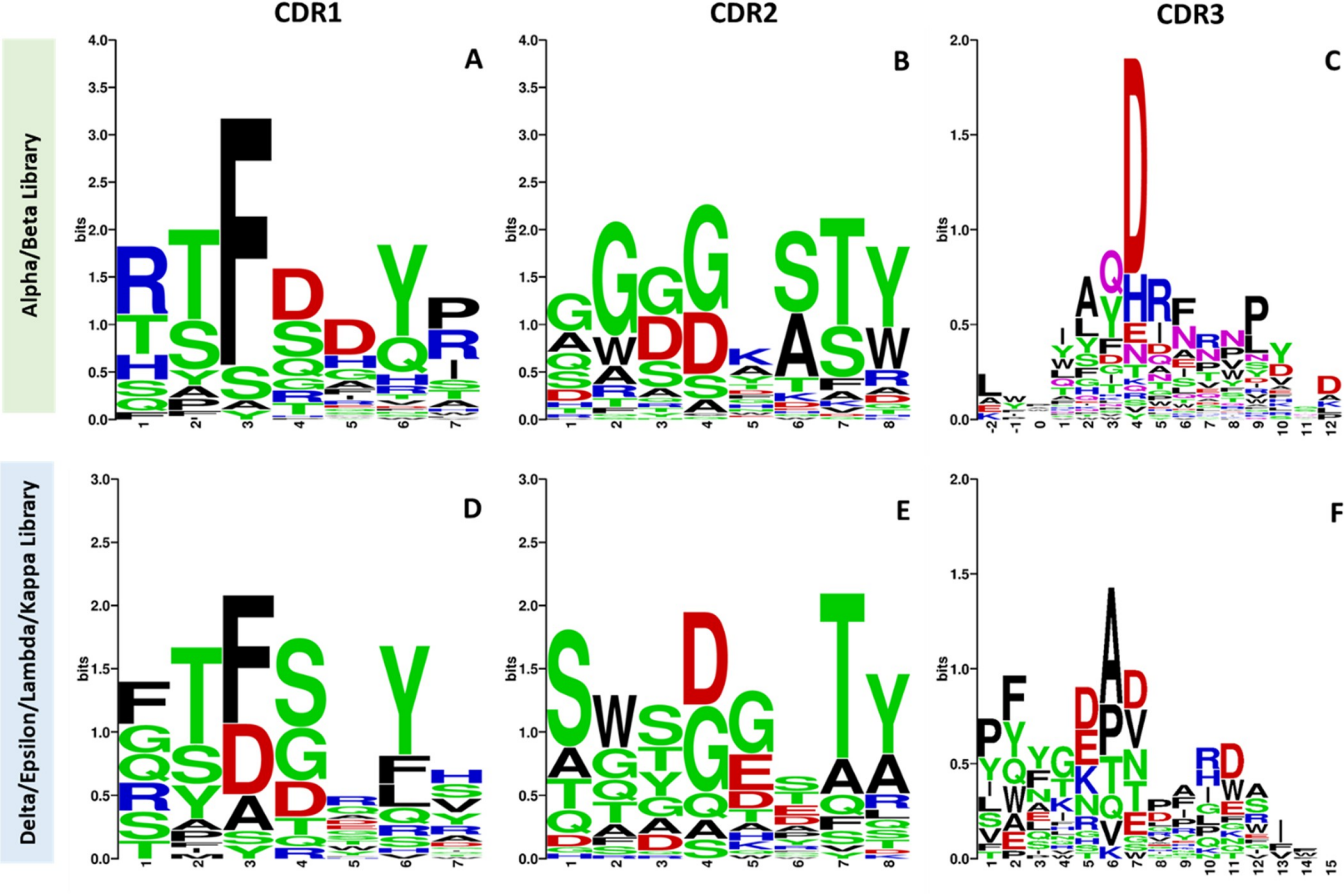
Two major clonotypes demonstrate affinity for groups of SARS-CoV-2 variants. The top sequence logos for CDR1, CDR2, and CDR3 are outputs of screening against the RBD from the Alpha and Beta variants Alpha/Beta (A, B, C). The bottom sequence logos for CDR1, CDR2, and CDR3 are the final sequences obtained after 4 rounds of screening against RBDs from SARS-CoV-2 variants Delta, Epsilon, Lambda, and Kappa (D, E, F). The sequence logo of the CDRs was aligned using the MUSCLE program and visualized using WebLogo, version 3. Bits represent the relative frequency of amino acids.

Four rounds of selection were used to identify clones that bind to the RBD region of SARS-CoV-2 variants. Single colonies were selected and used to generate clonal phage, which were expanded in deep-well 96 well plates. Phage-displayed Nbs were tested for RBD-binding by ELISA against the available Group 1 and Group 2 SARS-CoV-2 variant RBDs and were further tested for neutralization against SARS-CoV-2 variant pseudotyped vesicular stomatitis virus encoding an enhanced green fluorescent protein (eGFP) (VSV-SARS-CoV-2-GFP). Ninety-eight clones bound one or more SARS-CoV-2 variant RBDs and neutralized the corresponding VSV-SARS-CoV-2-GFP expressing the spike protein of the VOCs. The CDR sequences of these 98 clones were evaluated for common motifs which might be effective against sets of mutations in the SARS-CoV-2 RBD. Most of the diversity observed within the clonotype groups occurs in the complementarity determining region 3 (CDR3) which can be seen from the shift in positional diversity within each respective CDR **(Fig 2).**

**Fig 3:**
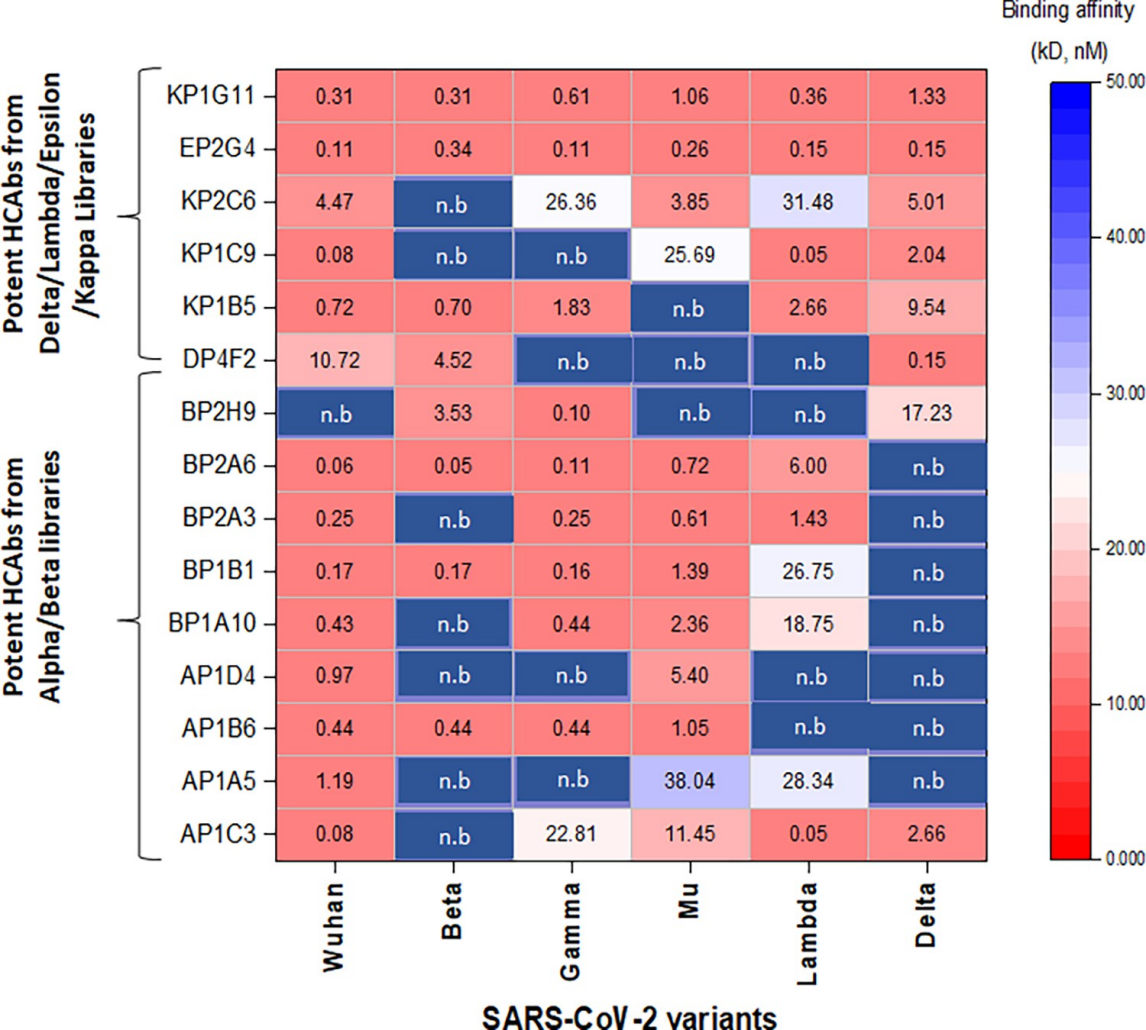
Summary of the binding affinity K_D_ (nM) of selected HCAbs against SARS-CoV -2 variant RBDs. A HCAb is considered non-binding (n.b) for variant RBDs when the respective K_D_ is > 125 nM. The data are the mean of triplicate experiments. To calculate the dissociation constants (K_D_) for each HCAb/variant combination, HCAb concentration–binding response data at Abs_450nm_ through ELISA were fit to the titration curve equation with a background term using a maximum likelihood estimation non-linear regression through curve-fitting program in Prism 9 (GraphPad Software, Inc.).

As previously described, the input library had a diversity of 3.2 x10^10^, with three possible CDR3 lengths. Sequencing of the input library showed that the 9-amino acid CDR3 was the most prevalent (40%), with 12-amino acid (34%), and 15-amino acid CDR3s (25%) comprising the rest of the library [18]. After screening against the VOC and VOI RBDs, the distribution of our top clones showed an increase in the prevalence of 9-amino acid CDR3s from 40% in the designed library to 69.5% in Alpha and Beta binders, and 51.4% in Delta, Lambda, Epsilon, and Kappa binders, whereas only around 8.5–10% of 15-amino acid length CDR3 were observed in enriched sequences **(S1A Fig).** Despite the diverse array of sequences, binding activity, and screened antigens, further analysis revealed that the amino acids that form these CDRs display remarkable conservation among output sequences targeting a functionally conserved epitope on various SARS-CoV-2 variant RBDs **(Figs 2, S1B and S1C)**. Importantly, hit clones have highly conserved CDR1s and CDR2s regardless of whether they bind the Group 1 or Group 2 RBDs (**Figs 2A, 3B, 3D and 3E**). As expected, the diversity responsible for differential Nb binding to different RBD groups is found in their CDR3s. For example, hit clones from screening against the Alpha and Beta RBDs shared an Asp residue at position 4 in CDR3 of the heavy chain (**Fig 2C**). Overall frequency of each amino acid across CDR3 loops of the viral variants was evaluated to characterize enrichment of certain amino acids across panning rounds. The frequency of Asp at position 4 was 70.1% in sequences with affinity for Alpha and Beta RBDs **(S1B Fig).** Similarly, the sequences with affinity for Delta, Lambda, Epsilon, and Kappa RBDs have a common motif with two amino acids with negatively charged side chains at positions 5 (52.4%) and 7 in CDR3 (44.3%) **(S1C Fig).**

From our 98 ELISA positives selected for sequencing, 59 sequences were identified as unique (S1 data). Humanized HCAbs were expressed as fusion proteins of Nbs with the crystallizable fragment (Fc) of human IgG1 for further characterization [27]. To determine how different sets of amino acid changes impact binding, all 59 HCAbs were evaluated for binding to Group 1 and Group 2 variant RBDs by titration ELISA **(S1 Table, Fig 3).** The top 37 candidates were selected based on their ability to bind one or multiple variant RBDs, and these were tested in neutralization assays against VSV-SARS-CoV-2-GFP expressing variant spikes **(S1 Table).** Of these, the top 15 HCAbs with high binding affinity and/or neutralization potency against multiple variant RBDs, were analyzed in kinetic and competition assays to characterize binding affinity and determine HCAb antigen epitopes. Almost all the HCAbs have high binding affinity for WT SARS-CoV-2 RBD **(Figs 3 and S2A).** The most promising HCAbs identified from screening against Alpha and Beta RBDs (notably AP1C3, AP2B6, BP1B1, BP2A6, and BP2H9) had sub nM binding affinity for similar Group 1 variant RBDs (Beta, Gamma, and Mu) but decreased binding affinity for Group 2 variant RBDs, particularly the Delta and Lambda lineages **(Figs 3 and S2B–S2F).** Similarly, HCAbs identified from screens against RBDs from Delta, Lambda, Epsilon, and Kappa variants (particularly KP1C9, KP2C6, and DP4F2) preferentially bind to Lambda and Delta RBD with little to no binding to Group 1 variant RBDs. In addition to group-specific binders, we did observe some HCAbs with cross-variant binding, including AP1C3 (with an affinity for RBDs from Mu, Lambda, and Delta variants), and EP2G4 and KP1G11, both with sub-nM K_D_ binding affinity against all RBDs in this study.

### Cross-variant HCAbs compete with ACE2 and neutralize SARS-CoV-2 variants

**Fig 4:**
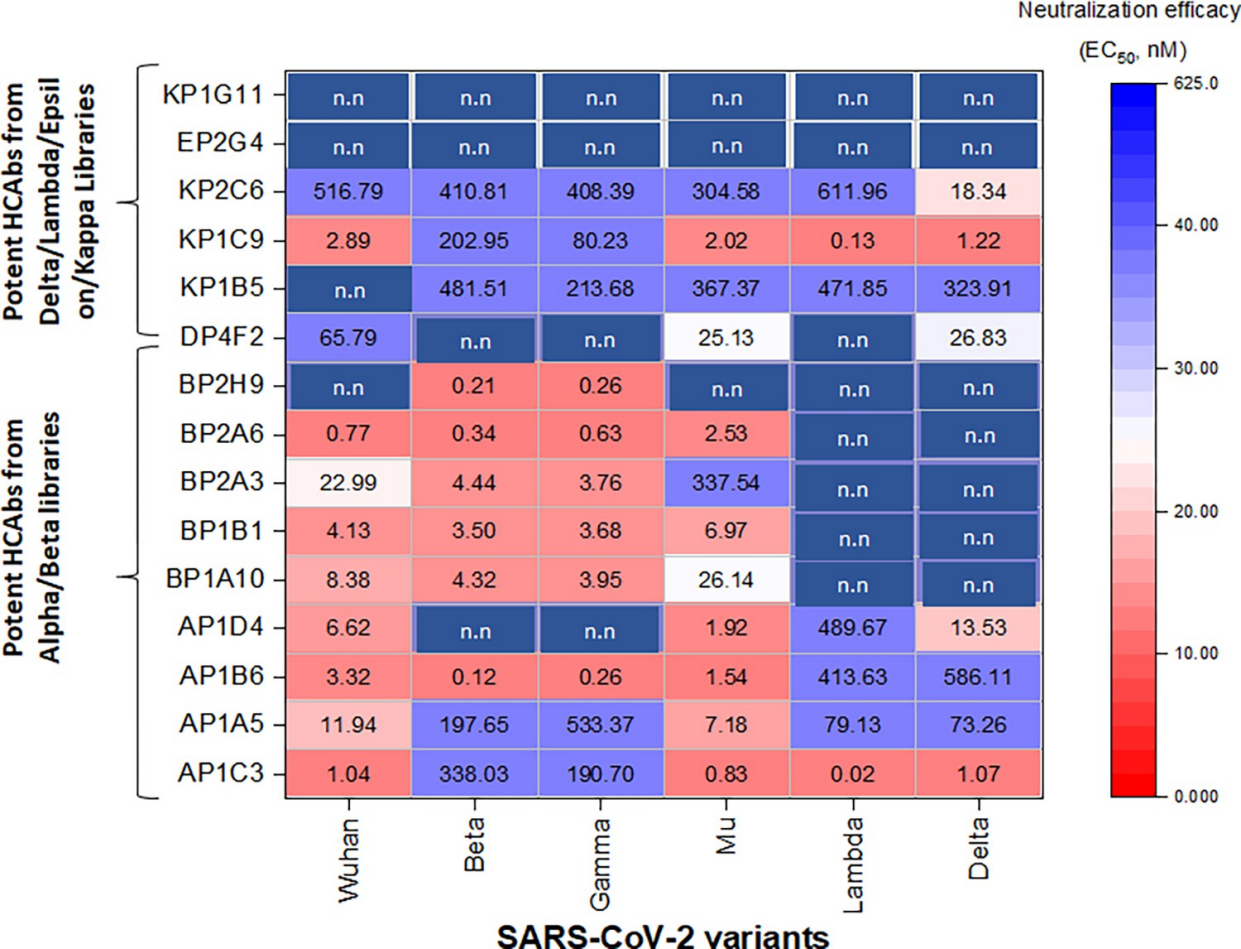
Summary of the neutralizing efficacy EC_50_ (nM) of selected HCAbs against VSV-SARS-CoV-2-GFP expressing variant Spikes. A HCAb is considered non-neutralizing (n.n) for a particular VSV pseudotyped virus variant when the respective EC_50_ is > 625 nM (50 ug/ml). The EC_50_ values were processed and fitted into nonlinear regression curve using GraphPad Prism 9.0. The data are the mean of triplicate experiments.

The top 37 HCAbs that demonstrated high binding affinity or cross-variant binding by ELISA to the VOI and VOC RBDs were tested for neutralization of VSV-SARS-CoV-2-GFP [28] expressing spike protein from the WT, Beta, Gamma, Delta, Lambda, and Mu variants **(S1 Table). Figs 4 and S3** summarize the neutralizing efficacy of selected HCAbs candidates against VSV-SARS-CoV-2-GFP [28] expressing variant spikes. Consistent with ELISA results, HCAbs identified from screening against Alpha/Beta variants (AP1B6, BP1B1, and BP2A6), potently neutralized VSV-SARS-CoV-2-GFP expressing WT and Group 1 spikes (Beta/Gamma), while outputs from screening against Lambda/Delta variants potently neutralized VSV-SARS-CoV-2-GFP expressing WT and Group 2 Spikes, including Delta. The most potent neutralizing HCAbs of the 37 tested were AP1B6, with a 50% effective inhibitory concentration (EC_50_) of 0.12 nM and 0.26 nM against Beta and Gamma pseudotyped variant viruses respectively, and AP1C3, with an EC_50_ of 0.02 nM and 1.07 nM against Lambda and Delta pseudotyped variant viruses respectively **(Figs 4, S3E and S3F).**

### Experimentally validated ML model enables rapid identification of the best heavy-chain only antibodies against new variants

The vast combinatorial mutational space of both antigens and antibodies presents an obvious challenge for antibody engineering and development, particularly when developing cross-variant antibodies or HCAbs. Here we tested whether an ML model could be trained and used to rapidly identify antibodies with efficacy against a wide range of mutants. Random forest models trained on Log_10_(K_D_) for HCAb binding to SARS-CoV-2 variant RBDs (Beta, Gamma Beta, Gamma, Delta, Lambda, & Mu variants) were generated and used to predict HCAb binding efficacy on novel variants. Features, namely, the Balaban- and Wiener-type index from Z, mass, van der Waals, electronegativity and polarizability weighted distance matrices, were derived from both the HCAbs CDRs, and from the predicted log escape fraction based on public datasets of conventional antibodies [29].

**Fig 5:**
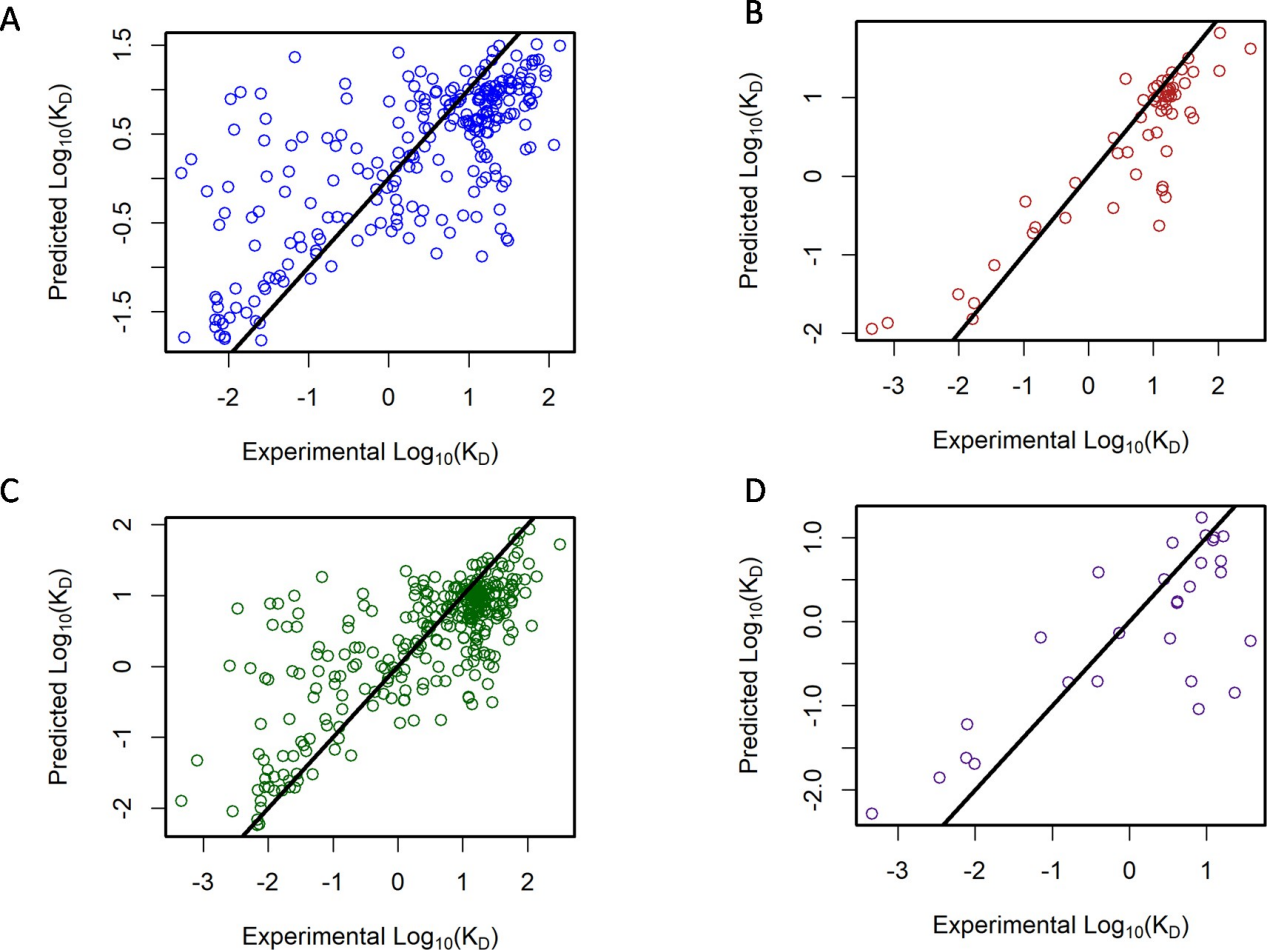
The relationship between predicted and observed HCAb dissociation constants for progressive stages of the iterative model building cycle as new variants are added. (A) A model trained for all pre-Omicron variants with five-fold cross-validation. (B) Using pre-Omicron variants as a training set and Omicron BA.1 as a test set. (C) Improved model including additional Omicron BA.1 data in training set and cross-validation. (D) Non-Omicron BA.5 used as the training set, and Omicron BA.5 as the test set.

The random forest models were then used to efficiently explore the functional impact of single critical mutations in the SARS-CoV-2 Beta, Gamma, Delta, Lambda, and Mu variant RBDs overcoming the experimental limitations of testing the combinatorically large numbers of potential mutations in Omicron BA.1 and Omicron BA.5. **Fig 5A** shows the five-fold cross-validation results for a random forest model trained only on pre-Omicron variants. This model had a root-mean-square error (RMSE) of 0.90 log_10_(μg/mL), Pearson correlation of 0.69, and Q^2^ of 0.47 **(Fig 5A).** Using these pre-Omicron variants as a training set and Omicron BA.1 as a test set yielded an RMSE of 0.57 log_10_(μg/mL), correlation of 0.9 (90% accuracy), and Q^2^ of 0.77 **(Fig 5B).** Interestingly, model performance for Omicron BA.1 binding prediction was better (**Fig 5B)** than what would be expected based on the cross-validation (**Fig 5A);** possibly due to the size of the test set. For the next iteration of the model, we added data for Omicron BA.1 to the training data (**Fig 5C**). The addition of BA.1 data to the training set resulted in an RMSE of 0.8 log_10_(μg/mL), correlation of 0.76, and Q^2^ of 0.58 (Fig 5C). Application of this random forest model to the BA.5 data yielded an RMSE of 0.86 log_10_ (μg/mL), correlation of 0.78 (78% accuracy), and Q^2^ of 0.58 **(Fig 5D).** Not surprisingly, addition of the Omicron BA.1 data resulted in significantly better results (Fig 5C vs **5A),** for two reasons: (1) inclusion of a larger training set and (2) addition of already well-predicted Omicron BA.1 data points to the test set. The predictions for the BA.5 variant shown in **Fig 5D** are well in line with what would be expected from the model result shown in **Fig 5C**.

**Fig 6:**
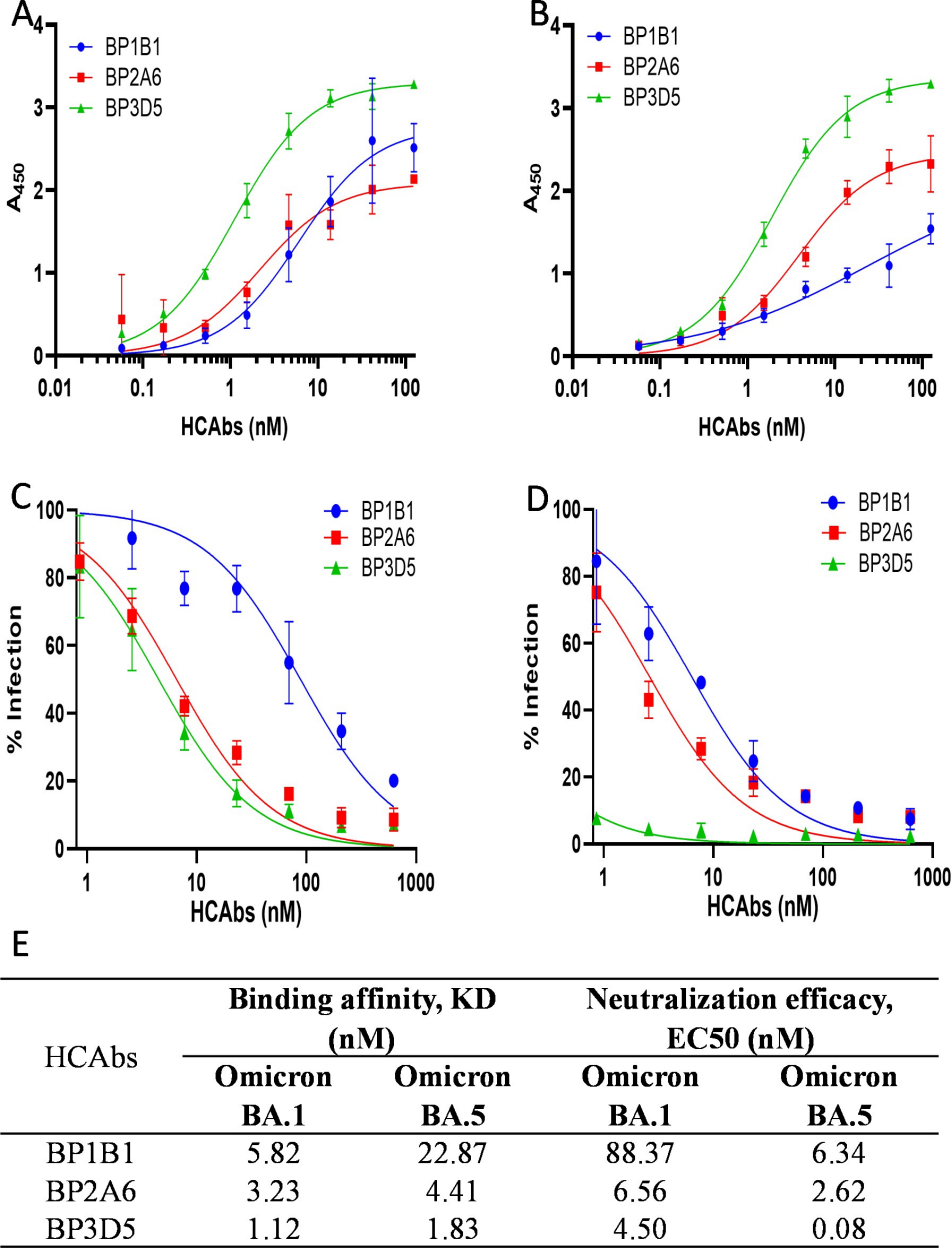
Characterization of HCAbs identified by ML model against Omicron BA.1 and Omicron BA.5. A, B: The binding affinity K_D_ (nM) of HCAbs against Omicron BA.1 (A) and Omicron BA.5 RBDs (B). C, D: The neutralizing efficacy EC_50_ (nM) of HCAbs against Omicron BA.1 (C) and Omicron BA.5 (D) pseudotyped variants. All experiments were performed in triplicate. E: Table summarizing binding affinity and neutralizing efficacy. To calculate the dissociation constants (K_D_) for each HCAb/variant combination, HCAb concentration–binding response data at Abs_450nm_ through ELISA were fit to the titration curve equation with a background term using a maximum likelihood estimation nonlinear regression through curve-fitting program in Prism 9 (GraphPad Software, Inc.). The EC_50_ values were processed and fitted into nonlinear regression curve using GraphPad Prism 9.0. All experiments were performed in triplicate.

The HCAbs identified as effective against Omicron using our ML model were experimentally validated and the best candidates against Omicron BA.1 and BA.5 were identified as BP1B1, BP2A6, and BP3D5. HCAbs were characterized through ELISA (**Fig 6A and 6B**) and neutralization assays with VSV-SARS-CoV-2-GFP expressing Group 3 (Omicron BA.1 and BA.5) spike variants **(Fig 6C and 6D).** All three HCAbs bound both Omicron variants, with BP3D5 displaying the highest binding affinity with a K_D_ of 1.12 and 1.83 nM against Omicron BA.1 and BA.5 RBD, respectively. BP3D5 was also the most potent in neutralization assays, with an EC_50_ value of 4.50 nM and 0.08 nM against VSV-SARS-CoV-2-GFP expressing Omicron BA.1 and BA.5 **(Fig 6E)**. BP3D5 was not one of the original top 37 HCAbs selected for neutralization assays prior to running the ML models. Once the ML-predicted BP3D5 was experimentally confirmed to be a strong binder and neutralizer of Omicron BA.1 and BA.5 we evaluated BP3D5 for neutralization of pseudotypes in group 1 and group 2. BP3D5 had neutralization activity against Group 1 pseudotyped viruses (Beta EC_50_ = 0.5 nM, Gamma EC_50_ = 1.4 nM, and Mu EC_50_ = 15.4 nM), but not against Group 2 pseudotyped viruses (Delta, Lambda) (**S1 Table** and S1, **S3** Figs)

**Fig 7:**
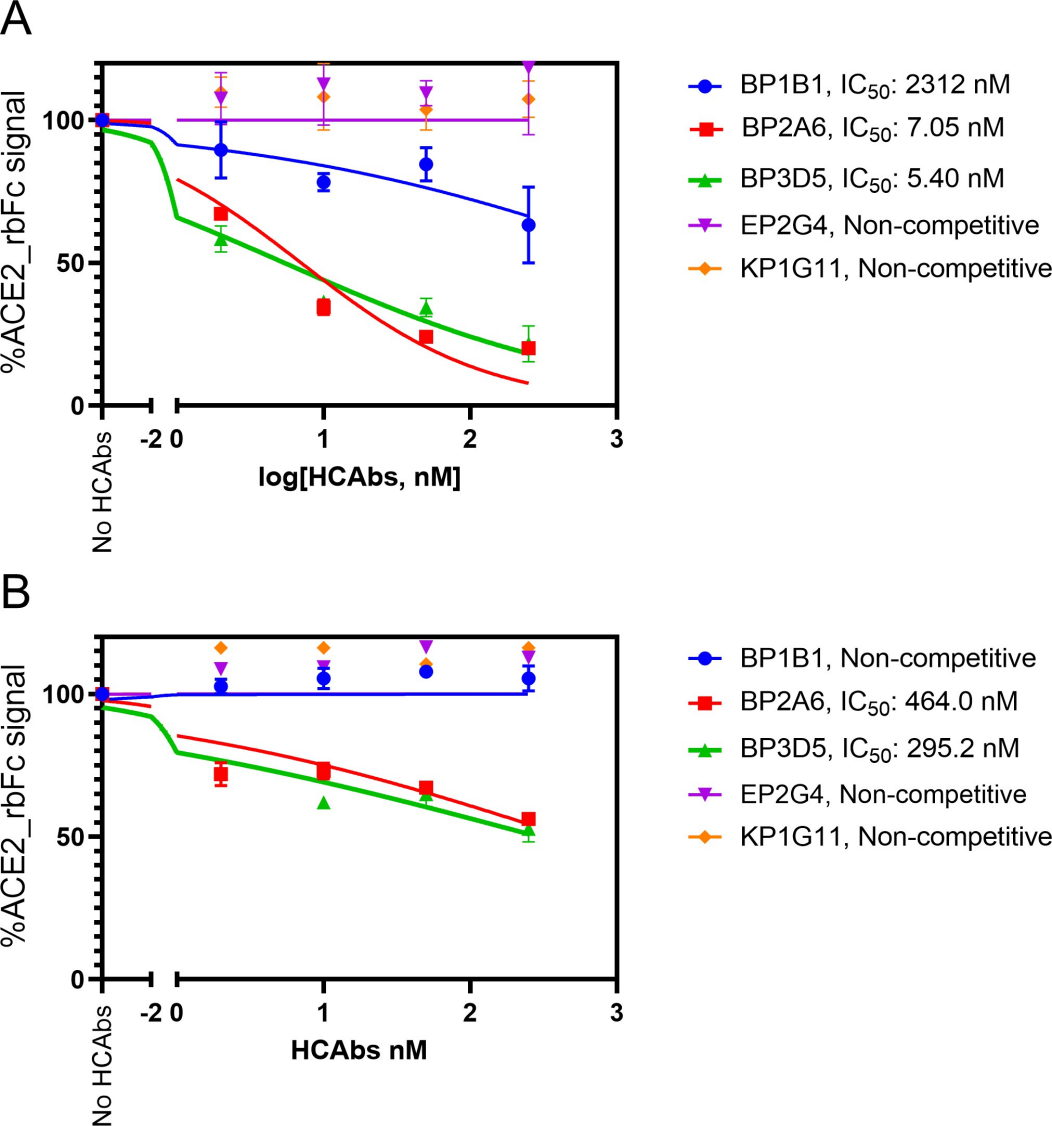
**HCAbs identified by ML model compete with ACE2-rbFc for binding to RBDs from Omicron BA.1 (A), and Omicron BA.5 (B).** Percentage ACE2_rbFc signal was calculated by dividing the maximum response in binding with SARS-CoV-2 RBD of the premixed ACE2_rbFc and HCAbs by the maximum response of ACE2_rbFc in solo binding of the SARS-CoV-2 RBD variant, multiplied by 100. The IC_50_ values were processed and fitted into nonlinear regression curve using GraphPad Prism 9.0. The experiments were performed in triplicate and the error is the standard deviation from the mean.

To further determine the mechanism of action, we performed competition assays between top candidate HCAbs and ACE2-rbFc (rabbit Fc domain) fusion protein [18] with competitive antibodies showing a decrease in ACE2 binding to RBD. Like their ability to block infection with VSV-SARS-CoV-2-GFP expressing Omicron BA.1, BP2A6 and BP3D5 were strongly competitive with BA.1 RBD binding to ACE2 (**Fig 7A**). BP2A6 and BP3D5 also blocked ACE2 binding to BA.5 but not to the same extent as with BA.1. BP1B1 did not seem to compete with ACE2 for binding to either BA.1 or BA.5 (**Fig 7A and 7B**), and may be decreasing infection by a non-competitive mechanism.

### Epitope binning and structure-based cross-docking analysis of HCAb–antigen interactions

**Fig 8:**
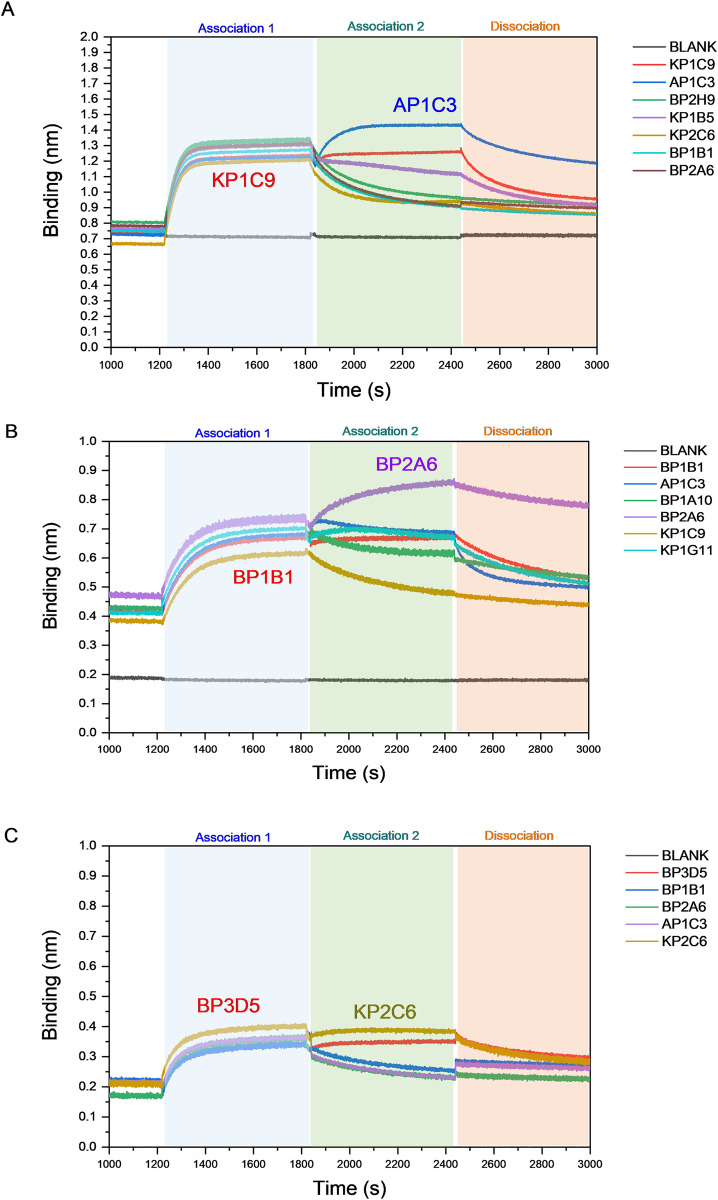
**Competition assay was conducted by using Bio-layer interferometry (BLI) to study binding against two epitopes on SARS-CoV-2 RBD variants, specifically Delta (A), Omicron BA.1 (B) and Omicron BA.5 (C).** Epitope binning was performed by injecting a first HCAb for 600 s, followed by injection of a second HCAb for 600 s, with a final dissociation step for 600 s.

Epitope binning by Bio-layer interferometry (BLI) was used to determine whether our top HCAbs had overlapping epitopes on SARS-CoV-2 RBD. Loading of KP1C9 onto Delta RBD did not seem to interfere with binding of AP1C3. **(Fig 8A).** However, when AP1C3 was loaded before KP1C9 it blocked binding of KP1C9 to Delta RBD, suggesting that while there is some overlap between the two HCAbs, AP1C3 can bind outside of the KP1C9 binding region (S4 Fig). Similarly, when BP1B1 was first bound to the Omicron BA.1 RBD it did not interfere with binding of BP2A6, But loading Omicron BA.1 with BP2A6 prevented binding of BP1B1 **(Figs 8B and** S4**).** In addition, binding of BP2A6 to Omicron BA.5 RBD prevented binding of BP3D5 to BA.5 suggesting that BP1B1 and BP3D5 have overlapping epitopes with BP2A6 but BP2A6 can bind outside of those overlapping regions. **(Fig 8C).**

**Fig 9:**
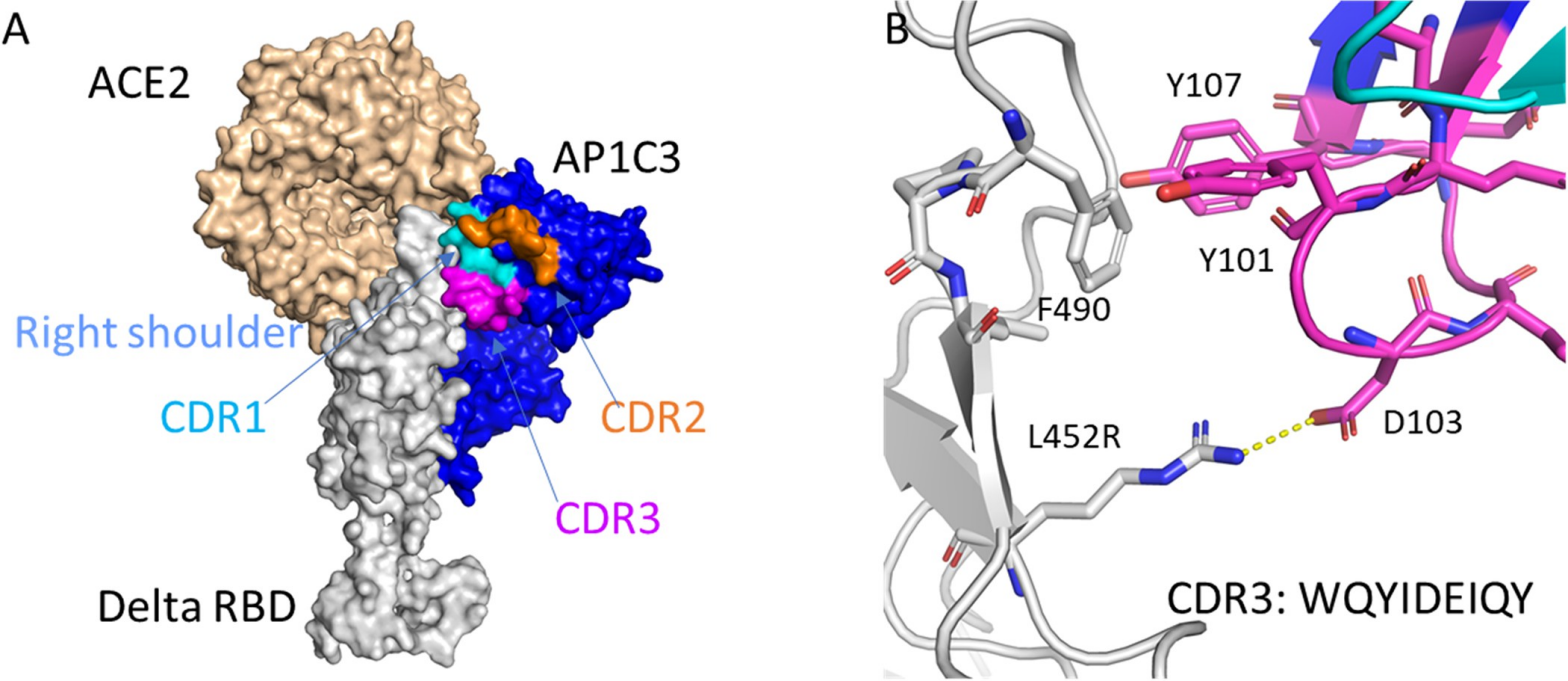
**Structural overlap of hACE2 with AP1C3: Delta RBD complex (A) and intermolecular interaction between RBD and AP1C3 at distinct epitopes (B).** Since these epitopes are among the least conserved regions on the spike protein, a critical point mutation (L452R) results in improved binding of AP1C3 with ultrahigh-affinity, resulting in potent neutralization by AP1C3.

To assess the potential to resist future mutation, we docked the HCAbs and hACE2 with Delta and Omicron BA.1 RBD variants to determine how the mutations in the Delta and Omicron variants impacted HCAb binding. In the Delta variant, there are two critical mutations in the RBD, L452R and T478K [30,31]. The L452R mutation has been reported to critically enhance the binding affinity to the host entry receptor ACE2, as well as transmissibility, fitness, and infectivity, and so improves viral replication [32,33]. In docking AP1C3 and ACE2 to the Delta RBD, we found that AP1C3 shares a strong electrostatic interaction that could compete with ACE2 in binding with L452R through electrostatic interaction at D103 **(Fig 9)**. The hydrophobic residue F490 (RBD) is clamped between two aromatic residues, Y101 and Y104 of AP1C3 which form a cation-π stacking interaction and contribute strongly to binding to the RBD and competition with ACE2.

**Fig 10:**
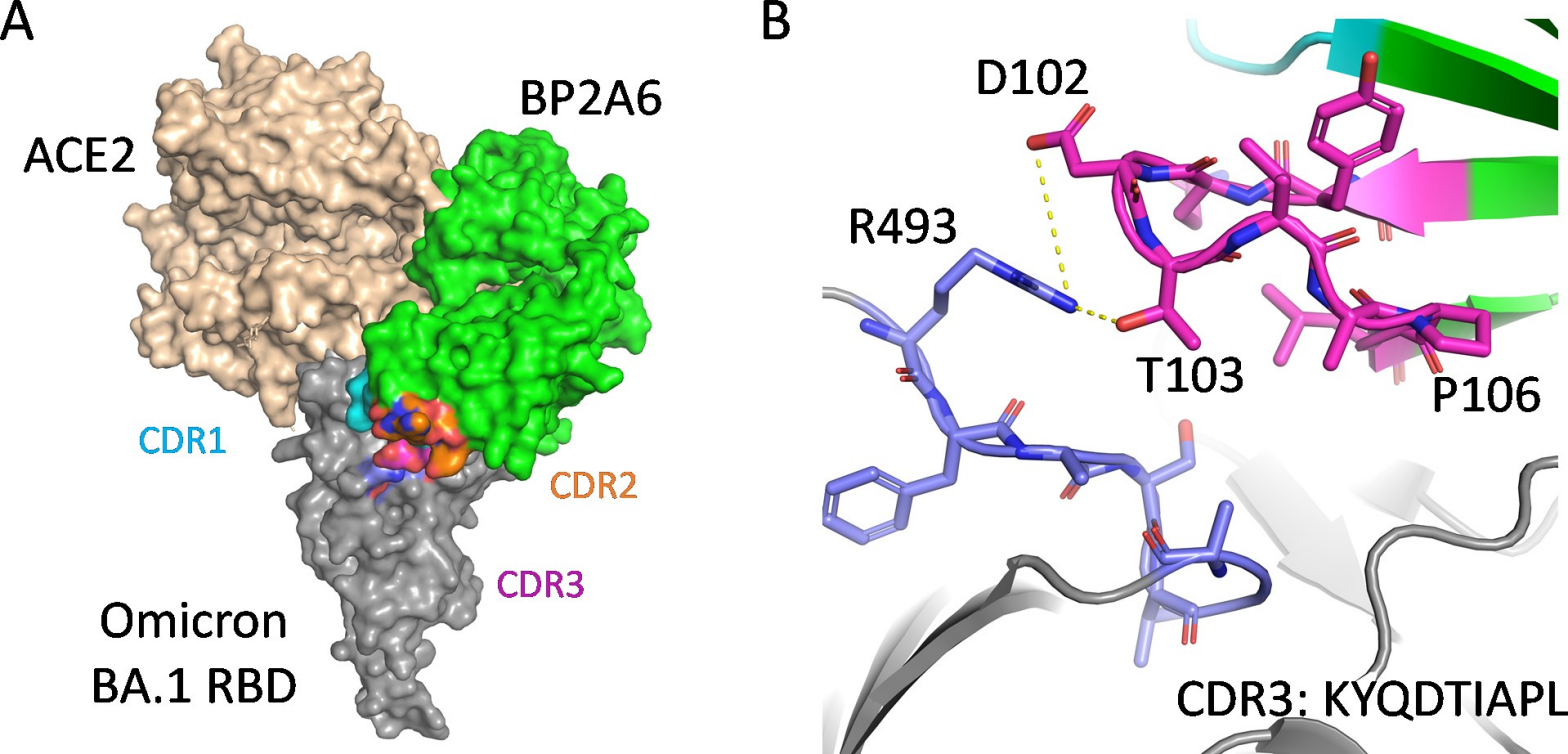
**Structural overlap of hACE2 with BP2A6: Omicron BA.1 RBD complex (A) and intermolecular interaction between RBD and BP2A6 (B).** Additional BP2A6: Omicron RBD complex interactions: side chains of Asp102, T103 (BP2A6) form hydrogen bonds with the main chain carbonyl groups of L492 and Y449 and the side chain of T470 (RBD), respectively. The main-chain carbonyl group of Asp102 (BP2A6) also forms a hydrogen bond with Q493R (RBD). Besides these polar interactions, A105, P106, and L107 of BP2A6 and Y483 of RBD form a cluster of hydrophobic interactions, providing ultrahigh-affinity and selectivity for RBD binding.

The HCAb BP2A6, selected by the ML model, showed multi-epitope binding to Omicron BA.1. The primary interaction between this antibody and the RBD surface is through a central residue, E484K, and its network of adjacent residues, including F490, F489, N487, F486, and V483. Notably, BP2A6 binds in an orientation that markedly differs from other neutralizing HCAbs in this study **(Fig 10B).** Its CDR3 adopts an extended beta-hairpin conformation that inserts into the receptor binding site (RBS) using both polar and hydrophobic interactions as well as bridging water molecules (Fig 1B). The flexibility of CDR3 is involved in a hydrophobic and aromatic patch that interacts with the RBD. Integrating these docking studies with HCAbs, RBD variants, and hACE2 interactions offers a comprehensive approach to understanding the antiviral mechanisms of these HCAbs and enables prediction of how potential mutations may impact efficacy.

To verify the docking result, we generated a set of RBDs with mutations in the residues identified by docking simulation. As expected, mutating the WT SARS-CoV-2 RBD at 452 from L to R increased binding to AP1C3, similar to AP1C3 binding to the Delta SARS-CoV-2 RBD which correlates with the docking results in Fig 9 **(S5A Fig).** While mutating F490 to H in the WT SARS-CoV-2 RBD reduced binding to AP1C3, The WT SARS-CoV-2 RBD containing F490M and F490V (other hydrophobic side chains) maintained higher binding to AP1C3 in comparison to WT SARS-CoV-2 RBD with no mutations, which suggested that a hydrophobic residue at 490 on RBD is critical for the higher binding of the Delta SARS-CoV-2 RBD to AP1C3 **(S5A Fig).**

For docking with Omicron BA.1 SARS-CoV-2 RBD, we created Omicron BA.1_R493Q and Omicron BA.1_R493E and compared their binding affinities with Omicron BA.1 SARS-CoV-2 RBD and WT SARS-CoV-2 RBD. BP2A6 binding to Omicron BA.1 RBD with the R493Q or the R493E mutation was lower than BP2A6 binding to WT SARS-CoV-2 RBD and Omicron BA.1 SARS-CoV-2 RBD, confirming the docking prediction that hydrogen bonds between BP2A6 and these residues are important for ultra high binding affinity **(S5B Fig).**

## Discussion

We successfully built an integrated high throughput experimental and computational pipeline to identify cross-variant neutralizing HCAbs. Of those identified, AP1B6, AP1C3, BP2A6, BP1B1, KP1C9, and BP3D5 are highlighted in this study because of their potent sub-nanomolar EC_50_s in preventing viral infection. The AP1B6, AP1C3, BP2A6, BP1B1, and KP1C9 constructs were identified from the experimental biopanning platform and required no affinity maturation to achieve potent neutralization against VOCs and VOIs (Figs 3–4). Notably, HCAbs AP1C3 and BP2A6 bind to two distinct regions on the Delta and Omicron BA.1 RBDs, respectively, from all other top candidate HCAbs (Fig 8), which suggests they could be used as part of a combination therapy to effectively prevent viral escape. To increase potency and reduce manufacturing costs, Nbs that bind to different antigen epitopes can be produced in multivalent formats

We also built upon previously published ML models that generalize and predict antibody binding against various viral strains [34,35]. Using generated efficacy data of HCAbs against existing variants, we built a sequence-based ML model of the dissociation constant of these HCAbs with Omicron BA.1 and Omicron BA.5. By using data from HCAbs, and varying their CDRs in the feature encoding method [14], we were able to limit the search space to identify potentially effective HCAbs more efficiently. We employed random forest models for exploring the functional impact of single critical mutations from the pre-Omicron data set, which provided a data-driven and efficient approach to predict how the complex mutations in Omicron BA.1 and Omicron BA.5 would impact HCAb efficacy. By performing a few minimal experiments to generate new data on more closely related variants, we were able to increase the accuracy of the model for both the Omicron variants tested (BA.1 and BA.5), but even with this additional data the accuracy of the model was lower for Omicron BA.5 (78%) than for Omicron BA.1 (90%). We hypothesize that Omicron BA.5 carries mutations that differ significantly from previous variants, and ML models trained on earlier data may not fully capture the specific characteristics and modelling impacts of these new mutations. Three HCAbs (BP1A1, BP2A6 and BP3D5) predicted to be effective against Omicron BA.1 and Omicron BA.5 by this ML model were experimentally validated using several assays. Neutralization activity was also determined for these three HCAbs against VSV-SARS-CoV-2-GFP expressing Omicron BA.1 or Omicron BA.5. Neutralization efficacy of BP2A6, and BP3D5 correlated well with inhibition of ACE2 binding to Omicron BA.1. BP1B1 did not seem to compete with ACE2 for binding to either Omicron BA.1 or BA.5 (**Fig 7A and 7B**), suggesting that BP1B1 causes a decrease in infection by an alternate mechanism. Our data demonstrates that continuous integration of experimental data to update and retrain ML models is essential to ensure that these models remain effective in predicting the behavior of newly emerging variants, resulting in better management of future pandemics and prevention of future surges.

## Materials and methods

### Protein production of SARS-CoV-2 variant RBDs

SARS-CoV-2 variant RBD plasmids and proteins (WT, Alpha, Beta, Gamma, Delta, Epsilon, Lambda, Kappa, Mu) were produced in the following way. These plasmids were constructed from ligation between the pSF-CMV plasmid and gBlocks of the SARS-CoV-2 variant RBD sequence (IDT), with edits made from the original SARS-CoV-2 RBD in the Snapgene software. Double-stranded DNA fragments encoding the SARS-CoV-2 variant RBD, an N-terminal Kozak sequence and signal peptide, and a C-terminal TEV cleavage site and 10xHisTag were commercially synthesized (IDT). The RBD DNA fragment and pSF-CMV plasmid, restriction digested with *NcoI* and *BamHI* were assembled using HiFi MasterMix (NEBuilder). The resulting plasmid was transformed into 10β *Escherichia coli* cells (NEB), amplified in overnight cultures, and purified using Miniprep (Epoch Life Sciences) and Midiprep kits (Qiagen).

SARS-CoV-2 variant RBDs were expressed in Expi293F cells (Thermo, A14527) in 50–100 ml transfections at 1 μg DNA/mL. Transfections were fed 18–22 hours later according to the manufacturer’s specifications and harvested six days post-transfection by centrifugation at 4000 xg for 20 min and filtering through a 0.22 μm filter (Nalgene). The filtered supernatant was run on a HisTrap Excel 5 ml Column (Cytiva) equilibrated in 20 mM phosphate, 300 mM NaCl buffer at pH 7.4 and subsequently through a Superdex 200 Increase 10/300 GL SEC column (GE) equilibrated in PBS. Purified proteins were concentrated using 10 kDa MWCO concentrators (Amicon) and quantified using the DC assay (Biorad). The biotinylated versions of the SARS-CoV-2 WT, Delta, Omicron BA.1 and Omicron BA.5 RBDs were purchased from Sino Biological.

### Phage preparation and purification

The phage display library was constructed as previously described by Stefan et al. (2021) [18]. The phage library for biopanning was produced from TG1 *E*. *coli* stock diluted to an initial OD_600_ of 0.1 in 2xYT-GA (31 g/L 2xYT, 100 mM glucose, 100 μg/mL Carbenicillin), and grown to an OD_600_ of 0.4–0.6 after which, CM13 phage was added to a final concentration of 2.0 × 10^9^ CFU/mL. The culture was incubated at RT for 15 min and then shaken at 37°C at 250 rpm for 30 minutes. *E*. *coli* were then pelleted at 5500 xg for 10 minutes, resuspended in 2xYT-AK (100 μg/mL Carbenicillin, 50 μg/mL Kanamycin), and incubated at 37°C overnight. To purify the phage, *E*. *coli* were pelleted at 5500 xg for 10 minutes at 4°C, and 1/4^th^ volume of 20% PEG, 2.5 M NaCl solution was added to supernatant containing phage. This solution was incubated at 4°C for 4 hours or overnight. The phage were then pelleted at 5500 xg for 1 hour at 4°C and resuspended in 10 mL PBS. The solution was centrifuged at 5500 xg for 5 minutes at 4°C 1–2 times further to pellet any remaining *E*. *coli*, after which a second 20% PEG precipitation step was performed on ice for 30 minutes. Finally, the phage was pelleted at 17900 xg for 10 min at 4°C, resuspended in PBS, sterile filtered through a 0.22 μm filter (Nalgene) and titered through serial dilution after incubation with TG1 *E*. *coli*.

### Biopanning

Four rounds (R1-4) of biopanning were performed against each SARS-CoV-2 variant RBD (Alpha, Beta, Delta, Epsilon, Lambda, Kappa). All rounds of panning were conducted in a 96-well plate format (Immulon 4 HBX Flat Bottom 1x12 strips). For the initial round 3 μg/well of the RBD was coated in each well using blocking buffer (100 mM NaHCO_3_, 150 mM NaCl; pH 8.3) overnight at 4°C; for subsequent rounds (R2-4), wells were coated with 2 μg/well of antigen as described above. In round 1, we coated 24 wells with 50 μl/well; in round 2–4, we coated 12 wells with 50 μl/well. Plates were extensively washed with PBST (0.05% Tween-20) on an EL 406 Plate Washer (Biotek). After washing, the block was added to each well for 2 hrs, which, depending on the round, was either Pierce Protein-Free Block (for R1; Thermo), 1% BSA in PBST (R2 & R4), or 2% milk in PBST (R3). Phage dilutions were made up in the corresponding block with either 2.5 × 10^12^ CFU/mL of the original Nb-phage library (R1) or 6.67 × 10^10^ CFU/mL of the previous round’s phage library for subsequent steps (R2-4); 150 μL of the phage dilution was added to the plate and shaken at 400 rpm at RT for 2 hours. The plate was washed with PBS and Tween percentages that were adjusted during different biopanning rounds as indicated in S2 Table.

To elute bound phage, 50 μL of 0.1 mg/mL trypsin (Sigma) in TBSC (10 mM Tris, 137 mM NaCl, 1 mM CaCl_2_; pH 7.4) was added to each well and incubated at 37°C for 30 minutes. Trypsin supernatant was collected, and this step was repeated for another 20 minutes. Log-phase TG1 *E*. *coli* was added to the pooled trypsin supernatant (3 mL of culture) and the trypsin-treated wells (100 μL per well). Both phage and *E*. *Coli* solutions were incubated at 37°C without shaking for 30 minutes, then shaking for another 30 minutes. Finally, the two preparations were combined, plated on 2xYT-GA plates, and titered as above. Colonies were scraped, resuspended in 10 mL 2xYT-GA, and regrown and purified as done above for the original phage library. After the final round, individual colonies were picked and grown in three Masterblock 2mL 96-well plates (Greiner) with CM13 infection, as done on the larger scale above.

A round of negative selection was carried out after the fourth panning round for additional screening and to identify further Nb candidates specific to a particular SARS-CoV-2 variant. Ten wells of a 96-well plate strip (Immulon) were coated overnight at 4°C with 2 μg/well of each variant SARS-CoV-2 RBDs except for the original panning antigen and structurally similar variants. As above, plates were washed with PBST using the Biotek plate washer and blocked with Pierce Protein-Free Block (Thermo) for 2 hours. Then 2.5 × 10^12^ CFU/mL (or if phage concentration was too low, undiluted) R4 phage library was added to row A and incubated shaking at 400 rpm for 1 hour. The phage supernatant was then transferred from row A to row B and incubated by shaking for 1 hour. This process was repeated for each variant RBD coated in a row of the plate until the phage supernatant was collected, used to infect TG1 *E*. *coli*, plated, and titered as above.

### Phage and heavy chain-only antibodies ELISAs

Nb-phage candidates were chosen based on performance in a monoclonal phage Enzyme-Linked Immunosorbent Assay (ELISA) against various SARS-CoV-2 variant RBDs, both before and after the negative selection round. Maxisorp Nunc 384-well plates were coated with 0.05 μg/well of each respective RBD in coating buffer overnight (100 mM NaHCO_3_, 150 mM NaCl; pH 8.3) at 4C. Plates were blocked with Pierce Protein-Free Block (Thermo) for 2 hours, after which 25μl of 1:10 dilution of phage preparation was added and incubated with shaking for 2 hours. Plates were washed as above, and mouse anti-M13 phage primary antibody (Invitrogen; 1:4000) was added for one hour of incubation. Plates were washed, and the HRP-Rabbit anti-mouse IgG secondary antibody (Abcam; 1:2000) was incubated for an additional hour. Finally, plates were washed and developed using TMB Ultra (Thermo). The reaction was stopped with equal volume 2 M H_2_SO_4_. Absorbance at 450 nm was monitored on a Tecan Spark plate reader.

Maxisorp Nunc 384 well plates were coated with 0.05 μg/well of SARS-CoV-2 variant RBD in coating buffer overnight at 4°C. Plates were washed as above between each step; wells were blocked with 50 μl/well Thermo Pierce Protein-Free commercial block for two hours. After washing, plates were incubated for 2 hours with primary Nb-huFc dilutions starting at 10 μg/ml and diluted 1:3 down the plate. Then, HRP-Goat anti-human IgG secondary antibody (1:15,000; Thermo) was added for 1 hour. Plates were developed with equal volumes of TMB and 2 M H_2_SO_4_. Absorbance at 450 nm was read on a Tecan Spark plate reader.

### Viruses and cell culture

VSV-ΔG-GFP pseudotyped viruses (except Omicron BA.1, Omicron BA.5) with VSV-G were produced in BHK-21 cells through transfection of pVSV-G at 20 μg DNA per 10cm plate and infection with ppVSV-reporter at an MOI of 0.1. The virus was harvested from the supernatant after centrifuging at 1000 xg for 10 min and filtering at 0.45 μm PES (GE). Using a serial virus dilution, VSV-ΔG-GFP was titered over Vero cells transfected with pVSV-G and incubated for 16–20 hours at 37°C. The SARS-CoV-2 variant spike plasmids were produced in the pCAGGS plasmid using gBlocks (IDT) for the matching spike variant. VSV-SARS-CoV-2 variant pseudo viruses (except Omicron BA.1, Omicron BA.5) were produced in Expi293 cells in 25-50ml transfections at 1 μg DNA per ml of culture of the respective pCAGGS plasmid form of the variant spike protein. Transfections were fed per the manufacturer’s instructions 18–22 hours post-transfection. At 72 hours post-transfection, Expi293 cells were infected with VSV-ΔG-GFP at an MOI of 3 for one hour, shaking at 37°C; cells were then pelleted at 1000 xg for 5 min and resuspended in fresh prewarmed Expi293 media supplemented with 0.137 μg/ml anti-VSV antibody (ATCC, from CRL-2700). At 18–24 hours post-infection, the cells were pelleted at 1000 xg for 10 minutes, and the viral supernatant was harvested, snap-frozen, and stored at -80°C.

A replication-competent vesicular stomatitis virus (VSV) expressing eGFP and the SARS-CoV-2 spike gene (rVSV-SARS-CoV-2-GFP) was provided by Dr. Sean Whelan for the Omicron BA.1 [36] and Omicron BA.5 variants. Replication-competent VSV chimeras were expanded in MA-104 cells in Dulbecco’s Modified Eagle medium (DMEM) supplemented with 2% fetal bovine serum (FBS), 100 units/mL penicillin, and 100 μg/mL streptomycin (Invitrogen, 15070063). Viral supernatants were harvested at the time of maximum cytopathic effect (approximately 48 hours post-infection), centrifuged at 1000 xg for 10 minutes to remove cellular debris, filtered over a 0.45 μm filter, and flash frozen with 10mM HEPES buffer. Virus aliquots were stored at -80°C. Viruses were titered by foci-forming unit assay over Vero-E6-TMPRSS-T2A-ACE2 cells. BHK-21 cells (Golden hamster kidney, ATCC CCL-10) were cultured in DMEM containing sodium pyruvate and supplemented with 10% FBS, 100 units/mL penicillin, and 100 μg/mL streptomycin. Vero cells (African green monkey kidney, ATCC CCL-81) were maintained in minimum essential medium alpha (αMEM) supplemented with 10% FBS, 100 units/ml penicillin, and 100 μg/ml streptomycin. Vero E6-TMPRSS-T2A-ACE2 cells (BEI NR-54970) were maintained in DMEM supplemented similarly to the above with 10 μg/ml puromycin. MA-104 cells (African green monkey kidney epithelial, ATCC CRL-2378.1) were maintained in DMEM with 10% FBS, 100 units/mL penicillin, and 100μg/mL streptomycin. All mammalian cell lines were grown at 37°C in 5% CO_2_.

### Foci-forming unit assays

Serial dilutions of virus aliquots were prepared in triplicate over Vero E6-TMPRSS-T2A-ACE2 cells in a 384-well format, with 25μL of dilution added per well. Cells were incubated at 37°C for 18 hours before fixation in 4% PFA. Fixed cells were counterstained with DAPI nuclear stain. Whole wells were imaged on a Tecan Spark Cyto plate reader, and the number of infected cells was determined. Viral FFU titers were determined as (number of infected cells)/(dilution factor*volume of dilution per well (mL)).

### VSV-SARS-CoV-2-GFP fluorescent reporter neutralization assays

Neutralization assays were carried out using confluent Vero cells (or Vero E6-TMPRSS2-T2A-ACE2 cells for Omicron subvariants) plated in black, coated, clear-bottom 384-well-plates (Corning) in supplemented αMEM (or supplemented DMEM for Omicron). Neutralizing HCAbs were serially diluted from a final concentration of 50 μg/ml; VSV-GFP virus (either rVSV-SARS-CoV-2-GFP or variant VSV-SARS-CoV-2-GFP) was added to a final MOI of 0.2. HCAbs and virus were incubated at 37°C shaking at 120 rpm for 30 min to 1 hour, and added to confluent cells. At 16–20 hours later, the plate was washed with PBS, fixed with 4% paraformaldehyde (Electron Microscopy Sciences), 1 μg/ml Hoechst 33342 nuclear stain (Invitrogen, H3570) solution for 30 min in the dark, rewashed, and imaged using the DAPI and FITC channels to visualize nuclei and GFP-positive cells on a Tecan Spark Cyto multi-mode plate reader and image cytometer. Images were analyzed using the Spark Control Image Analyzer Software. Fluorescence values were normalized using no-treatment infection values and dose-response curves, and EC_50_ values were generated using GraphPad Prism 9.

### Gyros HCAb-ACE2 competition assay

Competition assays between top candidate HCAbs and ACE2-rbFc (rabbit Fc domain) fusion protein [18] were carried out on the Gyrolab xPlore system in Bioaffy 1000 HC CDs. Samples were diluted in RexxipA buffer (Gyros Protein Technologies) and run using the General PK program. Biotinylated SARS-CoV-2 variant RBDs (Delta (Sino), Omicron BA.1 or BA.5 (Acro)) were used as the capture reagent at 10 μg/ml. A seven-point dilution series of HCAb (starting at 20 μg/ml; diluted 1:5 down) was pre-mixed with ACE2-rbFc (0.2 μg/ml in all samples & blank), and AlexaFluor 647 goat anti-rabbit IgG (H+L) (2 μg/ml; Invitrogen) was used for detection.

### Epitope binning using bio-layer interferometry

Epitope binning assays were carried out on the Octet RED384 system. Samples were prepared in the same buffer and plate as above. Streptavidin biosensors (Sartorius) were used to load biotinylated SARS-CoV-2 variant RBDs (Sino Biological; WT, Delta, Omicron BA1) at 0.15 μg/ml (5 nM). Remaining unbound streptavidin was quenched with 10 μM biotin solution, and association readings were read for two analytes (25 nM) in sequence.

### Model antibody/RBD structures

Structures for the antibodies were homology modeled using the SWISS-MODEL web server [37], which has greater than 60% sequence identity to all target sequences, as a template. The protonation states of titratable residues were predicted using PROPKA at pH 6.0 [38,39]. Modeled structures were further refined using Molecular dynamic (MD) simulations performed using NAMD ver. 2.1 [40] and the CHARMM36 forcefield [41]. The models were solvated in a box of TIP3P water molecules that extended 10 Å from the surface of the protein structure. Counterions (Na^+^, Cl^-^) were added to achieve a NaCl concentration of 0.1 M. MD simulations were run with periodic boundary conditions at a constant temperature of 333.15 K [42,43]. Solvated proteins were energy minimized for 10 ps and then gradually heated to the desired temperature (333.15 K) over 300 ps. The system was then equilibrated at 333.15 K for 5 ns under an isothermal−isobaric ensemble (NPT), followed by a 50 ns production run in the canonical (NVT) ensemble. Snapshots were collected at 5 ps.

### Machine learning model

To calculate dissociation constants (K_D_) for each HCAb/variant combination, concentration-response data were fit to the titration curve equation with a background term using a maximum likelihood estimation nonlinear regression. A 4-degree-of-freedom t-distribution was used to model the error to improve robustness. A competing constant model was also fit, and if it had a lower AIC for a given HCAb/variant combination, the data was removed from the set. Out of 399 HCAb/variant combinations, 4 were removed. Log_10_(K_D_) were used as the endpoint for a random forest model [44]. HCAb heavy chain CDR’s were combined into one long sequence and used to generate features with protR [45]. 8 features were based on sequence-order-coupling numbers with a maximum lag of four, and 8 features were scales-based descriptors based on a selection of ten Dragon [46] topological descriptors (namely, the Balaban- and Wiener-type index from Z, mass, van der Waals, electronegativity and polarizability weighted distance matrices). The top two principal components and a maximum lag of 2 were used. Two features were based on a model for antibody log escape fraction built from a public dataset [47] in particular, we used its log escape fraction predictions for Cov2-A2050 and Cov2-A2082 for the given variant’s sequence.

## Supporting information

S1 Table Several HCAbs characterized by titration ELISA and neutralization assay against several SARS-CoV-2 variant RBDs. The data are from experimental conditions performed in triplicateAn HCAb is considered non-binding (n.b.) for a variant RBDs when the respective kD is > 125 nM (10 ug/ml). An HCAb is considered non-neutralizing (n.n.) for a particular VSV pseudotyped viral variant when the respective EC50 is > 625 nM (50 μg/ml). Some HCAbs were assigned as non-tested (n.t.) because they did not show binding affinity with their biopanned variants.
(XLSX)

S2 Table PBS and Tween percentages for different biopanning rounds. (XLSX)

S1 Fig Distribution of amino acid residue lengths of CDR3 loops across libraries (A). Amino acid frequencies by position in CDR loops of various libraries (B, C). We classified 5 amino acid families by side-chain properties, Hydrophobic side chain: amino acids A, V, I, L, M, F, Y, W; Positively charged side chain: R, H and K; Negatively charged side chain: D, E; Special cases: C, G and P.
(TIF)

S2 Fig Several HCAbs characterized by titration ELISA against SARS-CoV-2 variant RBDs. A) WT, B) Mu, C) Beta, D) Gamma, E) Lambda, and F) Delta. The data are from experimental conditions performed in triplicate; the error is the standard deviation from the mean.
(TIF)

S3 Fig Neutralization efficacy of highest affinity HCAbs against VSV-SARS-CoV-2 expressing variant spike proteins. (A), Mu (B), Beta (C), Gamma (D), Lambda (E), and Delta (F). Effectiveness is measured in a half-maximal effective inhibitory concentration EC_50_ (nM) and the data shown is normalized to the infection rate in the absence of the HCAbs. The data are from experimental conditions performed in triplicate, the error is the standard deviation from the mean.
(TIF)

S4 Fig Competition assay was conducted by using Bio-layer interferometry (BLI) to study binding against two epitopes on SARS-CoV-2 Delta (A), Omicron BA.1 (B) and Omicron BA.5 (C) RBDs. Epitope binning was performed by injecting a first HCAb for 600 s, followed by injection of a second HCAb for 600 s, with a final dissociation step for 600 s.
(TIF)

S5 Fig Titration ELISA of RBDs from WT, Delta, and Omicron BA.1 SARS-CoV-2 variants with single point mutations with AP1C3 (A) and BP2A6 (B). The data are from experimental conditions performed in triplicate; the error is the standard deviation from the mean.
(TIF)

S1 Data Raw data used to build graphs in the manuscript are summarized. (XLSX)

## References

[1] Sampath S, Khedr A, Qamar S, Tekin A, Singh R, Green R, et al. Pandemics Throughout the History. Cureus. 2021;13(9):e18136. Epub 2021/10/26. doi: 10.7759/cureus.18136 ; PubMed Central PMCID: PMC8525686. 34692344 PMC8525686

[2] Bhadoria P, Gupta G, Agarwal A. Viral Pandemics in the Past Two Decades: An Overview. J Family Med Prim Care. 2021;10(8):2745–50. Epub 2021/10/19. doi: 10.4103/jfmpc.jfmpc_2071_20 ; PubMed Central PMCID: PMC8483091. 34660399 PMC8483091

[3] Planas D, Saunders N, Maes P, Guivel-Benhassine F, Planchais C, Buchrieser J, et al. Considerable escape of SARS-CoV-2 Omicron to antibody neutralization. Nature. 2022;602(7898):671–5. Epub 2022/01/12. doi: 10.1038/s41586-021-04389-z . 35016199

[4] Cao Y, Wang J, Jian F, Xiao T, Song W, Yisimayi A, et al. Omicron escapes the majority of existing SARS-CoV-2 neutralizing antibodies. Nature. 2022;602(7898):657–63. doi: 10.1038/s41586-021-04385-3 35016194 PMC8866119

[5] Mannar D, Saville JW, Zhu X, Srivastava SS, Berezuk AM, Tuttle KS, et al. SARS-CoV-2 Omicron variant: Antibody evasion and cryo-EM structure of spike protein–ACE2 complex. Science. 2022;375(6582):760–4. doi: 10.1126/science.abn7760 35050643 PMC9799367

[6] Wang L, Wang Y, Zhou H. Potent antibodies against immune invasive SARS-CoV-2 Omicron subvariants. Int J Biol Macromol. 2023;249:125997. Epub 20230726. doi: 10.1016/j.ijbiomac.2023.125997 . 37499711

[7] Sifniotis V, Cruz E, Eroglu B, Kayser V. Current Advancements in Addressing Key Challenges of Therapeutic Antibody Design, Manufacture, and Formulation. Antibodies (Basel). 2019;8(2). Epub 2019/09/24. doi: 10.3390/antib8020036 ; PubMed Central PMCID: PMC6640721. 31544842 PMC6640721

[8] Lu RM, Hwang YC, Liu IJ, Lee CC, Tsai HZ, Li HJ, et al. Development of therapeutic antibodies for the treatment of diseases. J Biomed Sci. 2020;27(1):1. Epub 2020/01/03. doi: 10.1186/s12929-019-0592-z ; PubMed Central PMCID: PMC6939334. 31894001 PMC6939334

[9] Norman RA, Ambrosetti F, Bonvin A, Colwell LJ, Kelm S, Kumar S, et al. Computational approaches to therapeutic antibody design: established methods and emerging trends. Brief Bioinform. 2020;21(5):1549–67. Epub 2019/10/19. doi: 10.1093/bib/bbz095 ; PubMed Central PMCID: PMC7947987. 31626279 PMC7947987

[10] Weitzner BD, Jeliazkov JR, Lyskov S, Marze N, Kuroda D, Frick R, et al. Modeling and docking of antibody structures with Rosetta. Nat Protoc. 2017;12(2):401–16. Epub 2017/01/27. doi: 10.1038/nprot.2016.180 ; PubMed Central PMCID: PMC5739521. 28125104 PMC5739521

[11] Sivasubramanian A, Sircar A, Chaudhury S, Gray JJ. Toward high-resolution homology modeling of antibody Fv regions and application to antibody-antigen docking. Proteins. 2009;74(2):497–514. Epub 2008/12/09. doi: 10.1002/prot.22309 ; PubMed Central PMCID: PMC2909601. 19062174 PMC2909601

[12] Sircar A, Gray JJ. SnugDock: paratope structural optimization during antibody-antigen docking compensates for errors in antibody homology models. PLoS Comput Biol. 2010;6(1):e1000644. Epub 2010/01/26. doi: 10.1371/journal.pcbi.1000644 ; PubMed Central PMCID: PMC2800046. 20098500 PMC2800046

[13] Magar R, Yadav P, Barati Farimani A. Potential neutralizing antibodies discovered for novel corona virus using machine learning. Scientific Reports. 2021;11(1):5261. doi: 10.1038/s41598-021-84637-4 33664393 PMC7970853

[14] Muyldermans S. A guide to: generation and design of nanobodies. Febs j. 2021;288(7):2084–102. Epub 2020/08/12. doi: 10.1111/febs.15515 ; PubMed Central PMCID: PMC8048825. 32780549 PMC8048825

[15] Yang X, Yang S, Li Q, Wuchty S, Zhang Z. Prediction of human-virus protein-protein interactions through a sequence embedding-based machine learning method. Comput Struct Biotechnol J. 2020;18:153–61. Epub 2020/01/24. doi: 10.1016/j.csbj.2019.12.005 ; PubMed Central PMCID: PMC6961065. 31969974 PMC6961065

[16] Liu-Wei W, Kafkas Ş, Chen J, Dimonaco NJ, Tegnér J, Hoehndorf R. DeepViral: prediction of novel virus-host interactions from protein sequences and infectious disease phenotypes. Bioinformatics. 2021;37(17):2722–9. Epub 2021/03/09. doi: 10.1093/bioinformatics/btab147 ; PubMed Central PMCID: PMC8428617. 33682875 PMC8428617

[17] Dong TN, Brogden G, Gerold G, Khosla M. A multitask transfer learning framework for the prediction of virus-human protein-protein interactions. BMC Bioinformatics. 2021;22(1):572. Epub 2021/11/29. doi: 10.1186/s12859-021-04484-y ; PubMed Central PMCID: PMC8626732. 34837942 PMC8626732

[18] Stefan MA, Light YK, Schwedler JL, McIlroy PR, Courtney CM, Saada EA, et al. Development of potent and effective synthetic SARS-CoV-2 neutralizing nanobodies. MAbs. 2021;13(1):1958663. Epub 2021/08/05. doi: 10.1080/19420862.2021.1958663 ; PubMed Central PMCID: PMC8344751. 34348076 PMC8344751

[19] Bannas P, Hambach J, Koch-Nolte F. Nanobodies and Nanobody-Based Human Heavy Chain Antibodies As Antitumor Therapeutics. Front Immunol. 2017;8:1603. Epub 2017/12/08. doi: 10.3389/fimmu.2017.01603 ; PubMed Central PMCID: PMC5702627. 29213270 PMC5702627

[20] Rambaut A, Holmes EC, O’Toole Á, Hill V, McCrone JT, Ruis C, et al. A dynamic nomenclature proposal for SARS-CoV-2 lineages to assist genomic epidemiology. Nature Microbiology. 2020;5(11):1403–7. doi: 10.1038/s41564-020-0770-5 32669681 PMC7610519

[21] Jangra S, Ye C, Rathnasinghe R, Stadlbauer D, Alshammary H, Amoako AA, et al. SARS-CoV-2 spike E484K mutation reduces antibody neutralisation. The Lancet Microbe. 2021;2(7):e283–e4. doi: 10.1016/S2666-5247(21)00068-9 33846703 PMC8026167

[22] Collier DA, De Marco A, Ferreira IATM, Meng B, Datir R, Walls AC, et al. SARS-CoV-2 B.1.1.7 escape from mRNA vaccine-elicited neutralizing antibodies. medRxiv. 2021:2021.01.19. doi: 10.1101/2021.01.19.21249840

[23] CM B, J V, H J. Outbreak of SARS-CoV-2 Infections, Including COVID-19 Vaccine Breakthrough Infections, Associated with Large Public Gatherings MMWR Morb Mortal Wkly Rep 2021;70:1059–62. 10.15585/mmwr.mm7031e2external icon. 34351882 PMC8367314

[24] Liu H, Wei P, Zhang Q, Aviszus K, Linderberger J, Yang J, et al. The Lambda variant of SARS-CoV-2 has a better chance than the Delta variant to escape vaccines. bioRxiv. 2021. Epub 2021/09/01. doi: 10.1101/2021.08.25.457692 ; PubMed Central PMCID: PMC8404886 holds equity at NB Life Laboratory LLC. We do not have any financial relations with Pfizer-BioNTech, Moderna, Eli Lilly, or Regeneron. 34462744 PMC8404886

[25] Riemersma KK, Grogan BE, Kita-Yarbro A, Halfmann PJ, Segaloff HE, Kocharian A, et al. Shedding of Infectious SARS-CoV-2 Despite Vaccination. medRxiv. 2021:2021.07.31.21261387. doi: 10.1101/2021.07.31.21261387 PMC9555632 36178969

[26] Chia PY, Ong SWX, Chiew CJ, Ang LW, Chavatte JM, Mak TM, et al. Virological and serological kinetics of SARS-CoV-2 Delta variant vaccine breakthrough infections: a multicentre cohort study. Clin Microbiol Infect. 2022;28(4):612.e1–.e7. Epub 2021/11/27. doi: 10.1016/j.cmi.2021.11.010 ; PubMed Central PMCID: PMC8608661. 34826623 PMC8608661

[27] Brooke Nicole Harmon; Peter Riches McIlroy YKL, inventorSARS-CoV-2 variant nanobodies and constructs comprising such nanobodies. US2024.

[28] Case JB, Rothlauf PW, Chen RE, Liu Z, Zhao H, Kim AS, et al. Neutralizing antibody and soluble ACE2 inhibition of a replication-competent VSV-SARS-CoV-2 and a clinical isolate of SARS-CoV-2. bioRxiv. 2020. Epub 2020/06/09. doi: 10.1101/2020.05.18.102038 ; PubMed Central PMCID: PMC7263548. 32735849 PMC7332453

[29] Greaney AJ, Starr TN, Barnes CO, Weisblum Y, Schmidt F, Caskey M, et al. Mapping mutations to the SARS-CoV-2 RBD that escape binding by different classes of antibodies. Nature Communications. 2021;12(1):4196. doi: 10.1038/s41467-021-24435-8 34234131 PMC8263750

[30] Yadav PD, Mohandas S, Shete AM, Nyayanit DA, Gupta N, Patil DY, et al. SARS CoV-2 variant B.1.617.1 is highly pathogenic in hamsters than B.1 variant. bioRxiv. 2021:2021.05.05.442760. doi: 10.1101/2021.05.05.442760

[31] Hoffmann M, Hofmann-Winkler H, Krüger N, Kempf A, Nehlmeier I, Graichen L, et al. SARS-CoV-2 variant B.1.617 is resistant to bamlanivimab and evades antibodies induced by infection and vaccination. Cell Reports. 2021;36(3):109415. doi: 10.1016/j.celrep.2021.109415 34270919 PMC8238662

[32] Ozono S, Zhang Y, Ode H, Sano K, Tan TS, Imai K, et al. SARS-CoV-2 D614G spike mutation increases entry efficiency with enhanced ACE2-binding affinity. Nature Communications. 2021;12(1):848. doi: 10.1038/s41467-021-21118-2 33558493 PMC7870668

[33] Tchesnokova V, Kulasekara H, Larson L, Bowers V, Rechkina E, Kisiela D, et al. Acquisition of the L452R Mutation in the ACE2-Binding Interface of Spike Protein Triggers Recent Massive Expansion of SARS-CoV-2 Variants. Journal of Clinical Microbiology. 2021;59(11):e00921–21. doi: 10.1128/JCM.00921-21 34379531 PMC8525575

[34] Einav T, Ma R. Using interpretable machine learning to extend heterogeneous antibody-virus datasets. Cell Reports Methods. 2023;3(8):100540. doi: 10.1016/j.crmeth.2023.100540 37671020 PMC10475791

[35] LaMont C, Otwinowski J, Vanshylla K, Gruell H, Klein F, Nourmohammad A. Design of an optimal combination therapy with broadly neutralizing antibodies to suppress HIV-1. Elife. 2022;11. Epub 2022/07/20. doi: 10.7554/eLife.76004 ; PubMed Central PMCID: PMC9467514. 35852143 PMC9467514

[36] Alsoussi WB, Malladi SK, Zhou JQ, Liu Z, Ying B, Kim W, et al. SARS-CoV-2 Omicron boosting induces de novo B cell response in humans. Nature. 2023;617(7961):592–8. doi: 10.1038/s41586-023-06025-4 37011668

[37] Waterhouse A, Bertoni M, Bienert S, Studer G, Tauriello G, Gumienny R, et al. SWISS-MODEL: homology modelling of protein structures and complexes. Nucleic Acids Research. 2018;46(W1):W296–W303. doi: 10.1093/nar/gky427 29788355 PMC6030848

[38] Olsson MHM, Søndergaard CR, Rostkowski M, Jensen JH. PROPKA3: Consistent Treatment of Internal and Surface Residues in Empirical pKa Predictions. Journal of Chemical Theory and Computation. 2011;7(2):525–37. doi: 10.1021/ct100578z 26596171

[39] Søndergaard CR, Olsson MHM, Rostkowski M, Jensen JH. Improved Treatment of Ligands and Coupling Effects in Empirical Calculation and Rationalization of pKa Values. Journal of Chemical Theory and Computation. 2011;7(7):2284–95. doi: 10.1021/ct200133y 26606496

[40] Phillips JC, Braun R, Wang W, Gumbart J, Tajkhorshid E, Villa E, et al. Scalable molecular dynamics with NAMD. J Comput Chem. 2005;26(16):1781–802. Epub 2005/10/14. doi: 10.1002/jcc.20289 ; PubMed Central PMCID: PMC2486339. 16222654 PMC2486339

[41] Huang J, Rauscher S, Nawrocki G, Ran T, Feig M, de Groot BL, et al. CHARMM36m: an improved force field for folded and intrinsically disordered proteins. Nature Methods. 2017;14(1):71–3. doi: 10.1038/nmeth.4067 27819658 PMC5199616

[42] Feller SE, Zhang Y, Pastor RW, Brooks BR. Constant pressure molecular dynamics simulation: The Langevin piston method. The Journal of Chemical Physics. 1995;103(11):4613–21. doi: 10.1063/1.470648

[43] Leeuw SWd Perram JW, Smith ER Rowlinson JS. Simulation of electrostatic systems in periodic boundary conditions. III. Further theory and applications. Proceedings of the Royal Society of London A Mathematical and Physical Sciences. 1983;388(1794):177–93. doi: 10.1098/rspa.1983.0077

[44] Wright MN, Ziegler A. ranger: A Fast Implementation of Random Forests for High Dimensional Data in C++ and R. Journal of Statistical Software. 2017;77(1):1–17. doi: 10.18637/jss.v077.i01

[45] Xiao N, Cao DS, Zhu MF, Xu QS. protr/ProtrWeb: R package and web server for generating various numerical representation schemes of protein sequences. Bioinformatics. 2015;31(11):1857–9. Epub 2015/01/27. doi: 10.1093/bioinformatics/btv042 . 25619996

[46] Mauri A, Consonni V, Pavan M, Todeschini R. DRAGON software: An easy approach to molecular descriptor calculations. MATCH Communications in Mathematical and in Computer Chemistry. 2006;56:237–48.

[47] Greaney AJ, Starr TN, Gilchuk P, Zost SJ, Binshtein E, Loes AN, et al. Complete Mapping of Mutations to the SARS-CoV-2 Spike Receptor-Binding Domain that Escape Antibody Recognition. Cell Host Microbe. 2021;29(1):44–57.e9. Epub 2020/12/02. doi: 10.1016/j.chom.2020.11.007 ; PubMed Central PMCID: PMC7676316. 33259788 PMC7676316

