# Supplementary figures and images for: Nanobody screening and machine learning guided identification of cross-variant anti-SARS-CoV-2 neutralizing heavy-chain only antibodies
Source: PLoS Pathog. 2025 Jan 23;21(1):e1012903. doi: 10.1371/journal.ppat.1012903 (PMC11793827; doi:10.1371/journal.ppat.1012903)

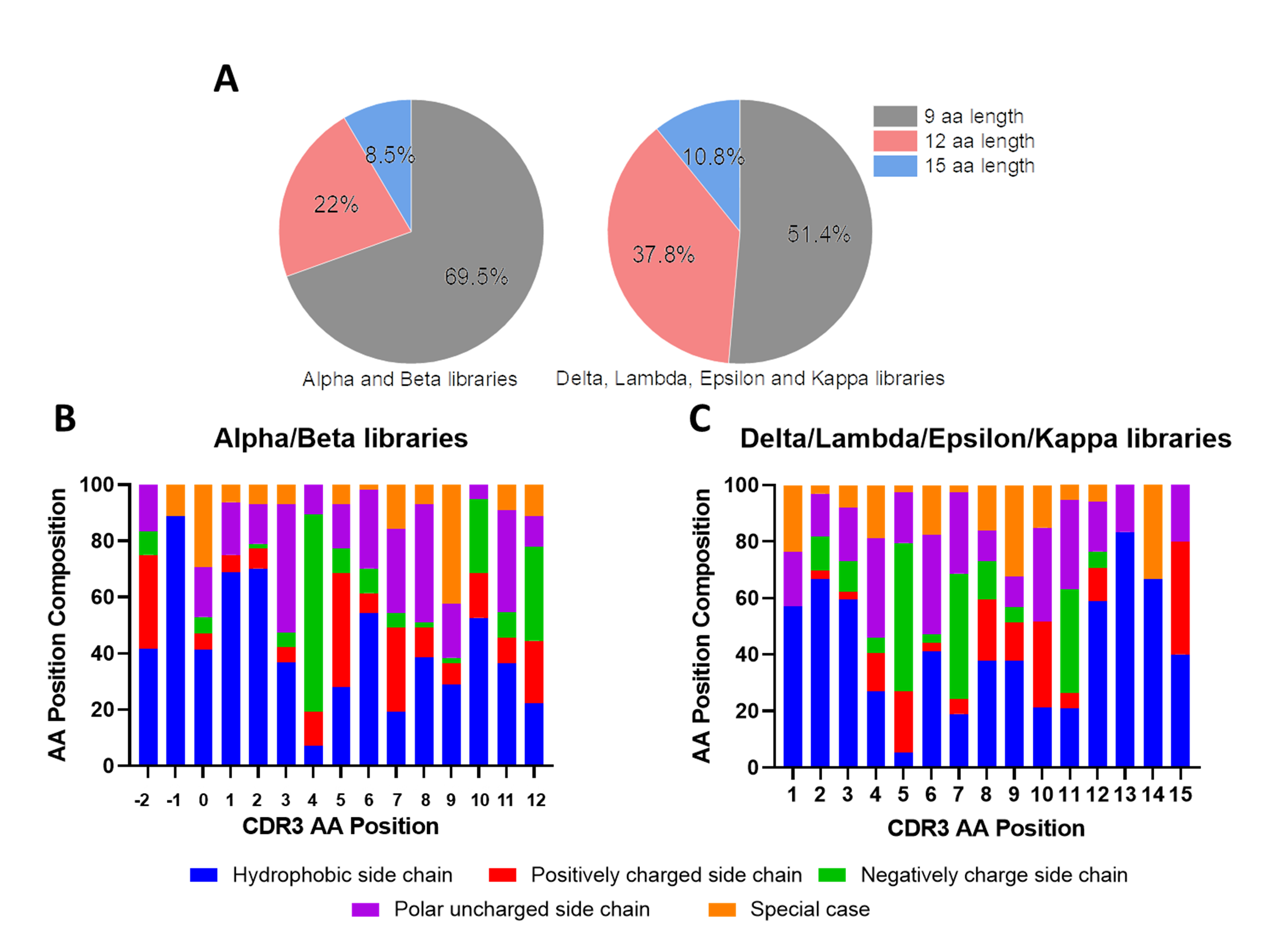

Supplement: S1 Fig — We classified 5 amino acid families by side-chain properties, Hydrophobic side chain: amino acids A, V, I, L, M, F, Y, W; Positively charged side chain: R, H and K; Negatively charged side chain: D, E; Special cases: C, G and P. (TIF) [file ppat.1012903.s003.tif]

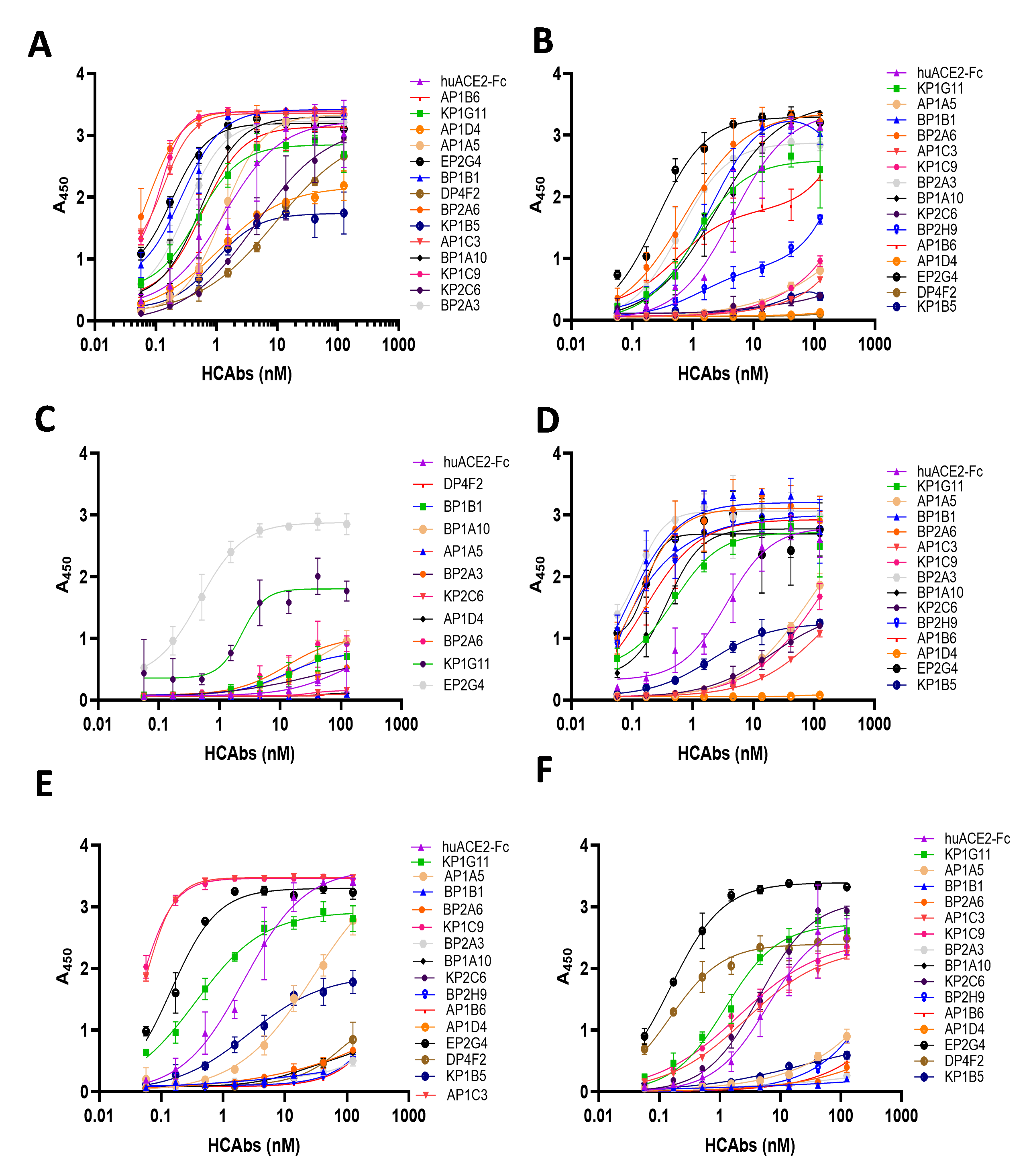

Supplement: S2 Fig — A) WT, B) Mu, C) Beta, D) Gamma, E) Lambda, and F) Delta. The data are from experimental conditions performed in triplicate; the error is the standard deviation from the mean. (TIF) [file ppat.1012903.s004.tif]

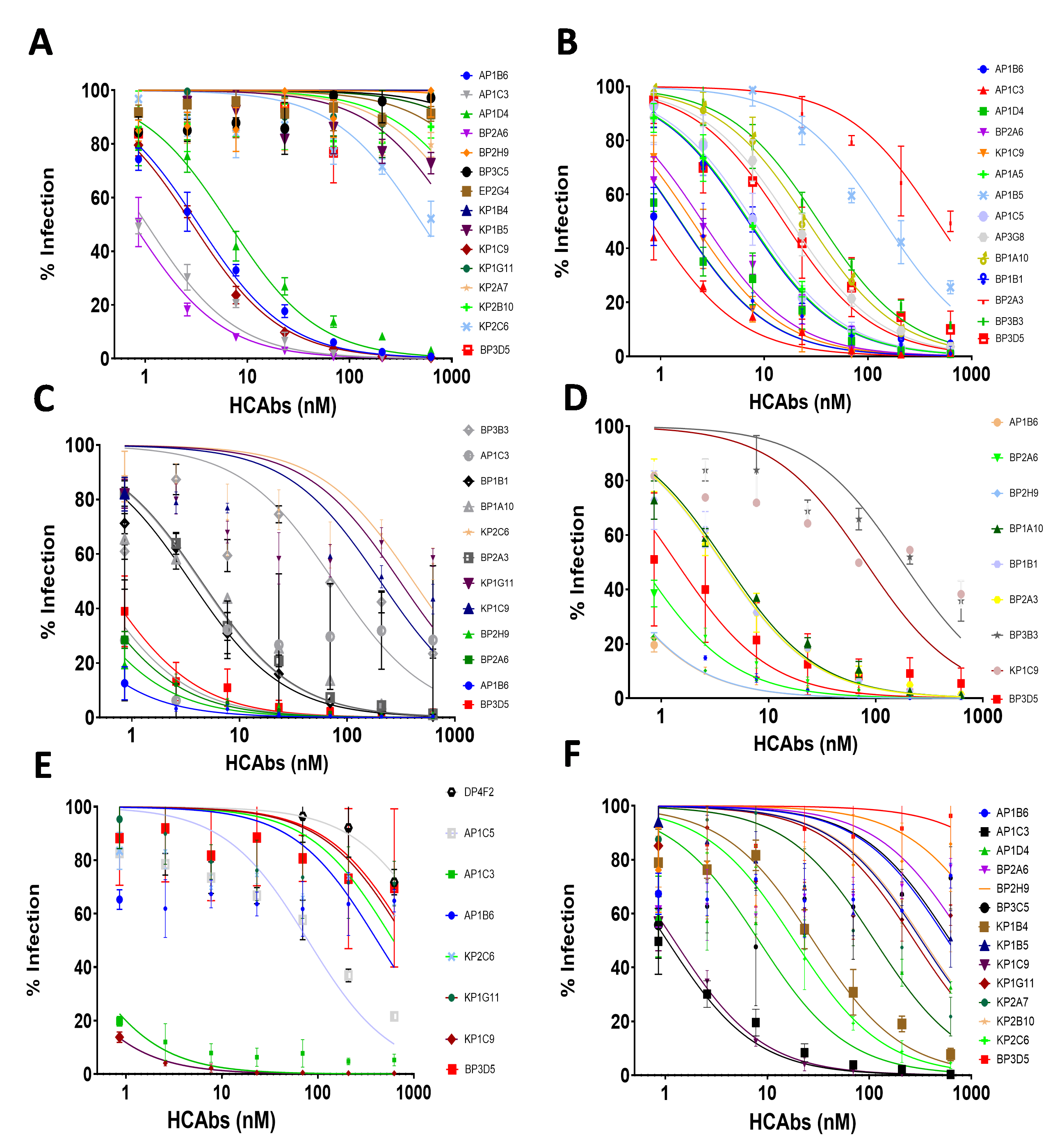

Supplement: S3 Fig — (A), Mu (B), Beta (C), Gamma (D), Lambda (E), and Delta (F). Effectiveness is measured in a half-maximal effective inhibitory concentration EC50 (nM) and the data shown is normalized to the infection rate in the absence of the HCAbs. The data are from experimental conditions performed in triplicate, the error is the standard deviation from the mean. (TIF) [file ppat.1012903.s005.tif]

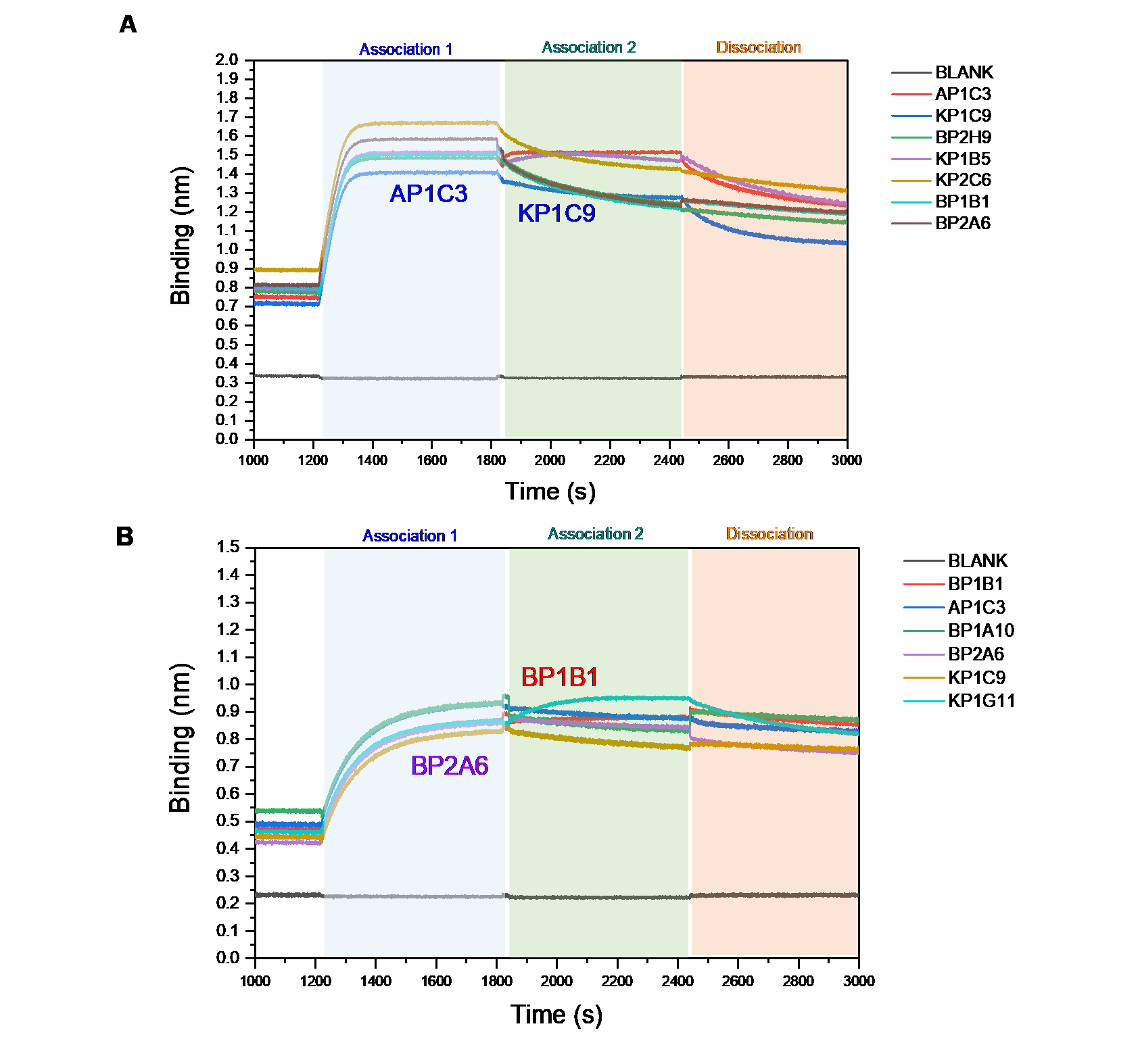

Supplement: S4 Fig — Epitope binning was performed by injecting a first HCAb for 600 s, followed by injection of a second HCAb for 600 s, with a final dissociation step for 600 s. (TIF) [file ppat.1012903.s006.tif]

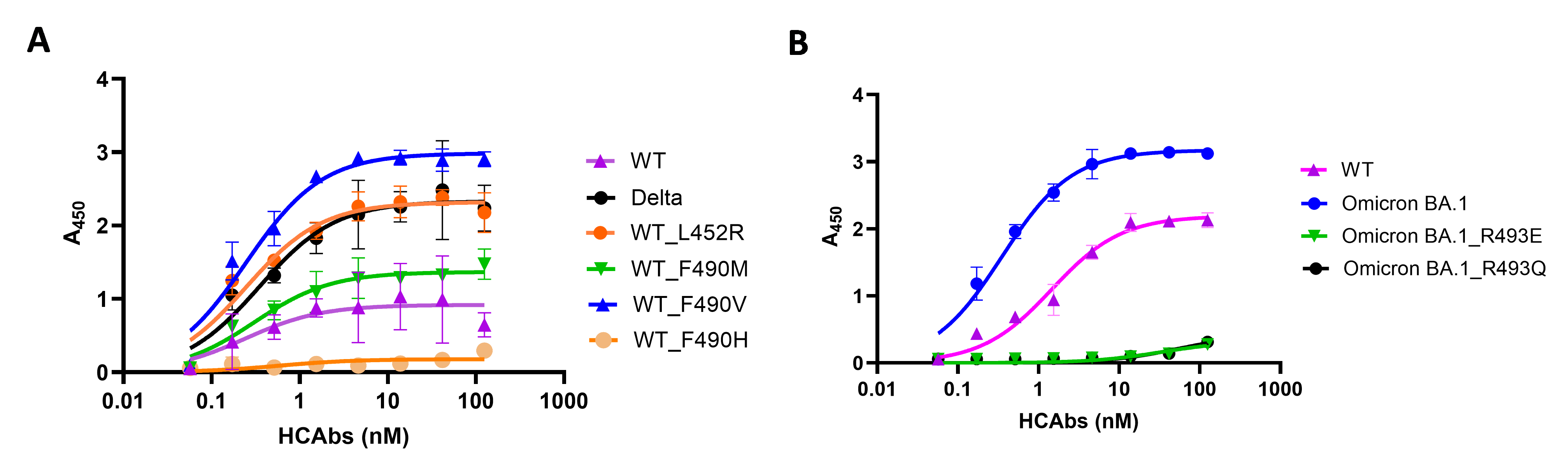

Supplement: S5 Fig — The data are from experimental conditions performed in triplicate; the error is the standard deviation from the mean. (TIF) [file ppat.1012903.s007.tif]
